# Inflammation and aging: signaling pathways and intervention therapies

**DOI:** 10.1038/s41392-023-01502-8

**Published:** 2023-06-08

**Authors:** Xia Li, Chentao Li, Wanying Zhang, Yanan Wang, Pengxu Qian, He Huang

**Affiliations:** 1 Bone Marrow Transplantation Center, the First Affiliated Hospital, Zhejiang University School of Medicine, Hangzhou, People’s Republic of China; 2 Liangzhu Laboratory, Zhejiang University Medical Center, 1369 West Wenyi Road, Hangzhou, 311121 China; 3 Institute of Hematology, Zhejiang University & Zhejiang Engineering Laboratory for Stem Cell and Immunotherapy, Hangzhou, 310058 China; 4 Zhejiang Province Engineering Laboratory for Stem Cell and Immunity Therapy, Hangzhou, 310058 China; 5 Zhejiang University-University of Edinburgh Institute, Zhejiang University School of Medicine, Zhejiang University, Haining, China; 6 Center for Stem Cell and Regenerative Medicine and Bone Marrow Transplantation Center of the First Affiliated Hospital, Zhejiang University School of Medicine, Hangzhou, 310058 China

**Keywords:** Inflammation, Senescence

## Abstract

Aging is characterized by systemic chronic inflammation, which is accompanied by cellular senescence, immunosenescence, organ dysfunction, and age-related diseases. Given the multidimensional complexity of aging, there is an urgent need for a systematic organization of inflammaging through dimensionality reduction. Factors secreted by senescent cells, known as the senescence-associated secretory phenotype (SASP), promote chronic inflammation and can induce senescence in normal cells. At the same time, chronic inflammation accelerates the senescence of immune cells, resulting in weakened immune function and an inability to clear senescent cells and inflammatory factors, which creates a vicious cycle of inflammation and senescence. Persistently elevated inflammation levels in organs such as the bone marrow, liver, and lungs cannot be eliminated in time, leading to organ damage and aging-related diseases. Therefore, inflammation has been recognized as an endogenous factor in aging, and the elimination of inflammation could be a potential strategy for anti-aging. Here we discuss inflammaging at the molecular, cellular, organ, and disease levels, and review current aging models, the implications of cutting-edge single cell technologies, as well as anti-aging strategies. Since preventing and alleviating aging-related diseases and improving the overall quality of life are the ultimate goals of aging research, our review highlights the critical features and potential mechanisms of inflammation and aging, along with the latest developments and future directions in aging research, providing a theoretical foundation for novel and practical anti-aging strategies.

## Introduction

Aging is a common, complex, and natural phenomenon. Aging research began in 1939 with the observation that restricting calorie intake could prolong life both in mice and rats.^1^ To further explain aging from the perspective of harmful inflammation and weakened immunity, inflammaging was introduced as an evolutionary perspective on immunosenescence, referring to the phenomenon of low-grade, chronic damage resulting from increased inflammation levels within the body.^2^ Later, inflammaging has been considered a hallmark of aging.^3^ Meanwhile, it is worth mentioning that can also damage the immune system, leading to immunosenescence during aging. For example, studies have shown that women living longer than men,^4^ in which older men showed higher activity of inflammation-related modules, with a more dramatic decrease in the ratio of naive T and B cells compared to older women.^4,5^ In addition, centenarians have been found to possess stronger anti-inflammatory abilities, suggesting that inflammation and immunity may a significant impact on the process of aging.^6,7^

**Fig. 1:**
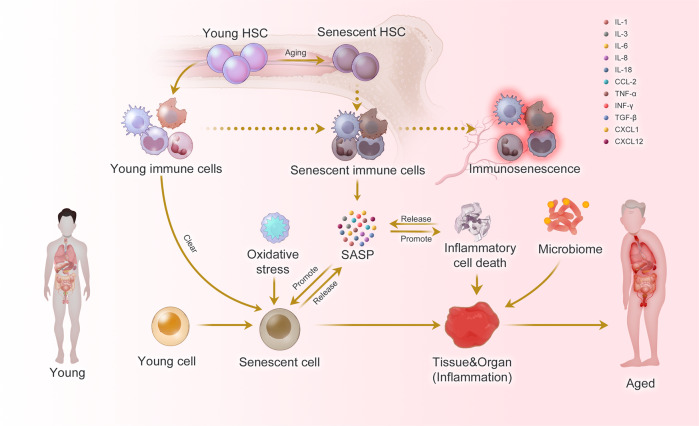
Inflammaging at the molecular, cellular, and organ levels. During the aging process, almost all cells in the body undergo senescence, a state characterized by a dysfunctional state and senescence-associated secretory phenotype (SASP). While immune cells play a crucial role in recognizing and eliminating these senescent cells, they are also affected by SASP, leading to a phenomenon called immunosenescence. Immunosenescence can impair the immunity to respond to infections and diseases, making the organism more vulnerable to illnesses. Moreover, the accumulation of senescent cells can trigger inflammation in organs, leading to organ damage and an increased risk of age-related diseases. This process is exacerbated by positive feedback loops that drive the accumulation of inflammation and organ damage, leading to further inflammation and an even higher risk of aging-related diseases

Considering the complexity of aging, multi-modal and multi-perspective studies are important. The process and accumulation of cellular senescence contribute significantly to the development of organ damage and diseases in organisms. Organ and organismal aging are often accompanied by the generation of inflammatory responses, and inflammation-associated molecular patterns promote cellular senescence, which in turn can lead to further inflammation, creating a vicious cycle (Fig. 1). In this review, we have discussed the concept of inflammaging across spatial and temporal scales, and complex factors leading to aging. We have also reviewed aging models, cutting-edge technologies in aging studies, and anti-aging strategies. Considering that preventing and alleviating the aging diseases and improving quality of life are the ultimate goals of aging research, our review shows current progress and directions in aging studies and provides a theoretical basis for new and feasible anti-aging strategies.

## Inflammaging at the cellular level

As the basic unit of the body, cellular senescence and the accompanying low-energy effects drive organismal aging. Recent studies have systematically summarized the biomarkers of cellular aging.^8,9^ Immunocytes, as key regulators of aging cells, have always been a focus of research due to their dysfunctional changes during aging.^10,11^ As early as 1969, Walford proposed “the immunologic theory of aging”,^12^ which further developed into the concept of immunosenescence, which is mainly manifested by a decrease of the body’s immune response to endogenous and exogenous antigens, leading to a decrease of the individual’s anti-tumor capacity and the ability to clear senescent cells (Fig. 1).^13^ Immunosenescence is a multifactorial cascade of events with different types of immune cells exhibiting different sensitivities.^14,15^ However, due to the inherent complexity of the mechanisms of immunosenescence, it is imperative to conduct research on immune cellular changes in multi-modal and systematic ways.

### Hematopoietic stem cells (HSCs)

**Fig. 2:**
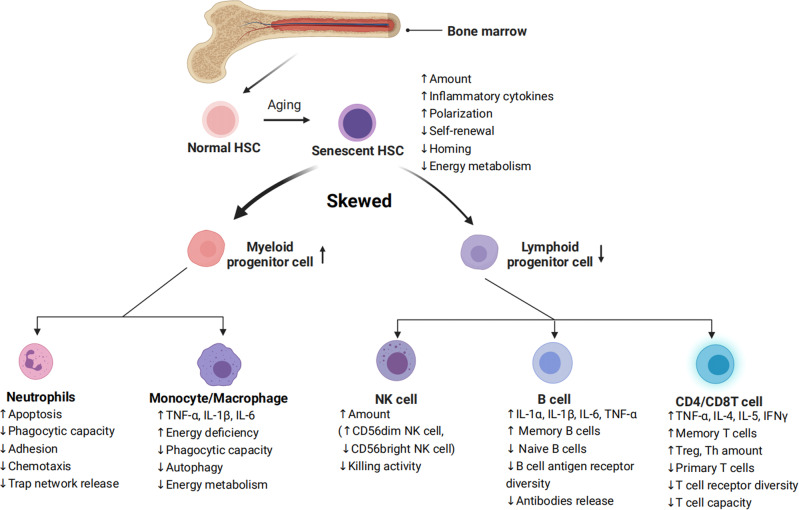
Characterization of HSC differentiation into immune cells during aging. Inflammation in senescent bone marrow impairs the function of HSCs. HSCs differentiate into various immune cells, and their senescence leads to changes in the number and functions of immune cells. Common features of immune cell senescence include a decline in performing immune functions and an increase in the release of inflammatory factors

Senescence of HSCs is the basis of immunosenescence. Senescent HSC differentiate into various types of dysfunctional immune cells, driving immunosenescence.^16^ Inflammation drives impaired self-renewal activity and accelerates aging of HSCs. Exposure to inflammatory stimuli during the early to mid-life stages in mice can lead to the eventual development of peripheral blood hemocytopenia, bone marrow (BM) cytopenia, and BM adipocyte accumulation, features that together constitute typical features of hematopoiesis in the elderly.^17^ The primary features of senescent HSC are characterized by changes in their self-renewal, differentiation bias, and energy metabolism (Fig. 2).

The increased myeloid/megakaryocytic differentiation bias is a major feature of senescent HSC.^18^ Numerous pro-inflammatory cytokines and growth factors, including granulocyte colony-stimulating factor (G-CSF), macrophage colony-stimulating factor (M-CSF), granulocyte-macrophage colony-stimulating factor (GM-CSF), interleukin (IL)-1, IL-3, IL-6, TNF-α, IFNs, and Flt3 ligands, have been found to promote the differentiation of hematopoietic stem/progenitor cells (HSPCs) towards myeloid cells over lymphoid cells, leading to imbalanced myelopoiesis and lymphopoiesis.^19–21^ For example, plasma cells that have accumulated in the bone marrow of aged mice can create a feedback loop of pro-inflammatory cytokines, such as IL-1 and TNF-α, which promote HSC myeloid differentiation bias.^22^ According to recent studies, bone marrow cells in senescent mice secrete more IL-1α/β,^23^ while damaged endosteum produces IL-1β to drive inflammation in the central bone marrow in trans to impede hematopoietic regeneration.^24^ When faced with in vivo stimulation by lipopolysaccharide (mimicking external microbial stimuli), senescent mice exhibited increased and prolonged IL-1α/β reactions.^23^ These illustrate IL1 as a key mediator of niche inflammatory damage to HSC. Conversely, neutralizing transforming growth factor (TGF) -β was found to reverse the age-related bias of HSCs towards megakaryocytic differentiation, leading to a greater generation of lymphoid progenitors and a more balanced lineage output of HSCs in transplantation experiments. In addition, inhibiting IL-6 improved the function of erythroid progenitors in aged mice.^25^ The results indicate that inflammaging is a key mediator of age-related HSC myeloid/ megakaryocyte differentiation biases.

HSC aging leads to a diminished capacity for self-renewal (Fig. 2), as shown by a significant increase in the number of mouse HSCs with age, but not a corresponding increase in the capacity of HSCs to undergo self-renewal. Studies in aged mouse HSCs have shown that older HSCs have overall reduced cell cycle activity.^26,27^ Notably, IFN-γ, a crucial pro-inflammatory cytokine, appears to have a dual role in regulating HSC proliferation. On the one hand, IFN-γ has been observed to stimulate HSC proliferation during infections.^28–30^ On the other hand, conflicting evidence has suggested that IFN-γ can hinder HSC regeneration by restricting self-renewal, rather than impacting quiescence or cell cycle progression.^30–33^

During inflammation, HSCs shift their energy metabolism from relying on anaerobic glycolysis to oxidative respiration.^34,35^ Accumulated reactive oxygen species (ROS) stress may trigger excessive DNA damage and HSC senescence and/or apoptosis.^36^ Binding to C-X-C motif chemokine ligand (CXCL) 12, C-X-C motif chemokine receptor (CXCR) 4 serves as a crucial mediator in numerous physiological and pathological processes, including inflammatory responses of the immune systems, regulation of hematopoiesis, induction of angiogenesis, as well as tumor invasion and metastasis.^37^ Mice with disrupted CXCR4 receptors experience a rise in endogenous production of ROS, which activates p38 mitogen-activated protein kinases (MAPK), triggers an increase in DNA double-strand breaks, and leads to apoptosis. As a result, there is a notable decline in the HSC repopulation potential.^37,38^ This depletion of HSC pools can be attributed to the elevated ROS levels, which are not related to the loss of quiescence in CXCR4-deficient HSCs. The CXCR4/CXCL12 axis has been found to limit apoptosis, DNA damage, and ROS elevation in HSCs by reducing mitochondrial oxidative stress.^38^ These findings suggest that inflammation could hasten the aging of HSCs and accelerate HSC functional decline (Fig. 2).

### Neutrophils

The role of neutrophils throughout the inflammatory response involves activation, migration, and clearance of pathogens and damaged tissues. The age-related decline in neutrophil function has a substantial influence on the development and advancement of various age-related diseases. Neutrophil development and numbers do not appear to be systematically altered with advancing age (Fig. 2).

Immunosenescence of neutrophils occurs in a low-grade inflammatory environment, with specific abnormalities in their metabolism and function, including decreased phagocytic capacity,^39^ abnormalities in adhesion and chemotaxis,^40,41^ increased apoptosis,^40,42^ abnormal neutrophil trap network release,^43^ and abnormal toll-like receptor function.^44^ In addition, as the organism ages, the transcriptomic and epigenomic profiles of neutrophils undergo significant remodeling.^45^

Past studies have focused on changes in neutrophils maintained in culture for a few hours in vitro, as they defined neutrophil senescence as its phenotypic change from release into the bloodstream to disappearance in the absence of inflammation. Another phenotypic change observed in neutrophils during in vitro culture is the downregulation of CXCR2 (CXCL1 receptor), a potent neutrophil chelator that has been shown to promote the release of neutrophils into the circulation and migration to sites of inflammation.^46,47^ In mice, senescence can disrupt the normal movement of neutrophils across epithelial layers in injured tissues through a CXCL1-mediated mechanism, resulting in abnormal neutrophil trafficking and consequential damage to distant organs.^48^ CXCL1 can also attract neutrophils to the liver of older mice, where they generate ROS and trigger tissue senescence and inflammation.^49^ Therefore, the role of neutrophils in defending against inflammation and pathogens is greatly weakened during aging (Fig. 2).

### Monocytes/macrophages

Apart from neutrophils, macrophages act as the initial responders to infections and participate in identifying, engulfing, and breaking down cellular debris and pathogens. The deterioration of macrophage function is a critical contributor to immunosenescence, where the capability of macrophages to effectively clear senescent cells from tissues reduces with aging (Fig. 2). Aged macrophages exhibit changes like reduced autophagy^50^ and a defect in their ability to fight viral infections.^51^

Aged macrophages display a noteworthy increase in SASP components, such as TNF-α, IL-6, and IL-1β. Furthermore, the ERCC1 gene deletion, which accelerates immune aging, was found to be responsible for the failure to excise the coding sequence for the DNA repair protein ERCC1 (ERCC1 gene deletion accelerates immune deficiency).^10^ Of particular importance, the anti-inflammatory cytokine IL-10 exhibited a decrease.^52^ This may lead to a tissue environment more prone to fibrosis, as IL-10 has been found to possess anti-fibrotic properties by suppressing pro-fibrotic molecules, including TGF-β.^53,54^ Besides, senescent macrophages show significant upregulation of both cell-cycle checkpoint inhibitors p16INK4a and p21CIP1 in a mouse model with deficiency in repairing DNA damage^10^ and downregulate both glycolysis and mitochondrial oxidative phosphorylation, which leads to an energy-depleted state that impairs the functioning of macrophages (Fig. 2).^55^

### Natural killer (NK) cells

NK cells are fundamental cells of the innate immune system and are regarded as the primary defense mechanism for human health. Recent findings indicate that NK cells play a central role in the immune surveillance of aging cells, and that dysfunctional NK cell activity is associated with infections, malignant tumors, inflammatory diseases, and an increased burden of aging cells with advancing age.^56^ While age does not seem to affect the number of NK progenitors in the peripheral blood or bone marrow,^57^ most studies suggest that the aging process causes an elevation of the overall number of NK cells in older adults.^58,59^ However, this increase in NK cell number is accompanied by a decline in their ability to proliferate and kill targets (Fig. 2).^60–62^ Specifically, there tends to be a decrease in the proportion of immature CD56 bright NK cells and an increase in the percentage of CD56 dim NK cells.^61,63^ CD56 dim cells produce many cytokines and mainly play an immunomodulatory role. They also account for more than 90% of NK cells, the majority of which are cytotoxic and have strong killing activity. Moreover, changes in the expression of NKp30, NKp46, and DNAM1 (NK activation receptors) in the elderly can impair the immune surveillance function of NK cells.^64–66^ Due to age-related functional decline, NK cells from younger donors exhibit a greater potential for expansion than those from older donors when subjected to in vitro stimulation with IL-2, underscoring the susceptibility of NK cells to age-related dysfunction.^61^ Also, the signs of reduced NK cell effector functions, such as decreased cytotoxicity, as well as lower expression of perforin and granzyme and reduced secretion of IFN-α and IFN-γ but more IL-1, IL-4, IL-6, IL-8, IL-10, and TNF-α are identified.^67,68^ Besides, with increasing donor age, the frequency of T cell precursors in CD34+Lin- cells tends to decrease, while the frequency of NK/T cell precursors tends to increase.^69^ This suggests that the lymphoid differentiation potential of peripheral blood precursor cells shifts from T cells to NK/T cells with age, meaning that more HSCs differentiate into NK/T cells. Meanwhile, a notable rise in the quantity of both NK and NKT cells occurs after the age of 60 (Fig. 2).^70^

### B cells

B cells always work as antibody producers have an essential role in immunity.^71^ Age-related changes in B cell composition are the main reason for decreased antibody response to vaccination and infection in older adults (Fig. 2). Lymphopoiesis of B cells continues during the life cycle. The output of B cells is severely affected by changes in the microecology of the bone marrow, such as decreased pro-B cell-survival cytokine IL-7 level.^72^ The number of B-cell precursors and antibody-producing plasma cells in mouse and human bone marrow decreases with age.^73^ Further, the proliferative potency of lymphoid progenitor is also impaired by ageing, while that of myeloid progenitor does not changes.^74^ Different from mice, as individuals age, there is a decline in the proportion and absolute number of B cells in the peripheral blood.^75,76^ Especially, the aging process is associated with a rise in the proportion of late-stage exhausted memory B cells,^77^ while the percentage of memory B cells that exhibit a positive correlation with influenza vaccine responses significantly decreases with age.^70,78^ Furthermore, the number of B cells mobilized after antigenic stimulation is only 1/10 to 1/50 that of normal adult animals in the elderly. Similarly, the seropositive protection rate in those aged 60-74 years after influenza vaccination was 41% to 58%, decreasing to 29% to 46% for those 75 years or older. Meanwhile, a collapse in B cell diversity has been discovered.^79^ However, the elderly tend to exhibit an increase in autoantibodies, which can elevate the risk of developing autoimmune diseases.^80^

B cells not only produce antibodies, but also play regulatory effector functions in the development of memory T-cells (Fig. 2). Memory B cells are more prevalent in older adults and can produce various pro-inflammatory cytokines and chemokines such as IL-1α, IL-1β, IL-6, and TNF-α, suggesting their potential involvement in inflammatory disorders during inflammaging.^81^ Moreover, aging mice exhibit increased frequencies of age-associated B cells (ABCs) in their bone marrow, which secrete higher levels of TNF-α, a cytokine that impairs the generation of young pro-B cells.^82^ This observation suggests that bone marrow-resident ABCs may contribute to altered B cell development with age.

### T cells

As fighters of pathogens, their dysfunction makes the mice less resistant to infection and get muscle atrophy. These dysregulated T cells even release many inflammatory molecules to accelerate aging,^83^ which emphasizes the role of T cells in aging. As a crucial immune cell type, T cell replenishment is achieved by the export from the thymus and self-renewal of peripheral naive T cells. In general, CD4 T cells are adaptable to the challenges of aging and keep naive-memory imbalance to a minor level. Compared with CD4 naive cells, the naive-memory imbalance in CD8 T cells is considerable. A decline in the number of circulating naive CD8 T cells is the most significant and consistently observed marker of immunosenescence in healthy older adults. Like CD4 T cells, BATF/IRF4 also promotes the transformation of naive CD8 T cells to effector CD8 T cells, which upregulates transcription factors related to effector functions, including T-bet, Runx3, and Blimp-1.^84^

With aging, the number of T helper cells (Th) and T regulatory cells (Treg) increases. The levels of cytokines secreted by Th1 and Th2 cells diminishes with age, making the body less able to defend itself against external pathogens. Elderly individuals exhibit increased expression of TGF-β receptor 3 (TGFβR3) on naive CD4 cells. This leads to the activation of a transcription factor network that includes PU.1, BATF, and IRF4, ultimately resulting in a preference for Th9 differentiation.^85,86^ The increased Th9 leads to the increased secretion of the signature cytokine IL-9, which mediates various inflammatory responses and is involved in the differentiation of autoimmune diseases and inflammatory diseases.^85^ Although Treg cells increase in number with age, their suppressing capability declines significantly, which may contribute to inflammation in the elderly.^87,88^ At the molecular level, the damage to signal transduction, such as decreased CD28-mediated JNK kinase and Raf-1/MEK/ERK kinase activation, results in a hypo-responsiveness of T cell receptor (TCR) signal transduction.^87^ Meanwhile, effector memory CD45RA (EMRA) CD8 T cells show significant SASP, including high levels of IL-18 and disintegrin and metalloproteinase 28 (ADAM28, a proteinase involved in the cleavage of membrane-bound TNF-α).^89,90^ However, it is noteworthy that the cell cycle of EMRA CD8 T cells is partially reversible, which is different to senescent T cells.^91^

In old people, highly differentiated T cells, especially memory T cells display the loss of co-stimulatory molecules such as CD27/28, representing an earlier stage of senescence or exhaustion.^92,93^ Exhausted T cells display several hallmarks similar to aging ones, such as mitochondrial dysfunction^94^ and epigenetic dysregulation.^95^ Traditionally, it is believed that Exhausted T cells lack the function of secreting inflammatory, anti-inflammatory, and cytotoxic effector molecules.^96^ However, Denis et al. have recently substantiated that exhausted GZMK-expressing CD8 T cells can accelerate the inflammatory phenotypes.^97^

In the peripheral blood lymphocyte subsets of healthy adults in different ages, it was found that the decreased naive CD4 and CD8 T cell number, increased memory CD4 or CD8 T cell number, and decreased CD28 expression on T cells.^70^ Numerous studies have shown a close association between increased stimulation by various antigens in vitro, especially cytomegalovirus (CMV) infection, and an increase in effector memory T cells,^98^ resulting in the activation of naive lymphocytes into memory lymphocytes and their long-term presence in vivo.^13,99^ This process leads to an increased number of memory CD4 and CD8 T lymphocytes with age, and a decrease of TCR diversity in naive T cells, which suppresses the responsiveness of T cells to neoantigens (Fig. 2).

In summary, as the body ages, most immune cells exhibit senescent characteristics, which manifests internally as difficulty in clearing senescent/damaged cells and externally as weakening of the body’s resistance.

## Inflammaging at the organ level

As a result of the effects of cellular senescence, chronic inflammation, and immunosenescence, the pathological aging of organs increases the level of inflammation and makes repair difficult, ultimately leading to diseases.^10^

### Lymphoid organs

The primary lymphoid organs, including the bone marrow and thymus, are responsible for immune cell development. However, with advancing age, these organs undergo a functional decline, which results in compromised capability of replenishing the immune cell reservoir. Senescence of the lymphatic organs promotes immunosenescence and plays a key role in organ inflammaging.

#### Aged bone marrow promotes HSC-related immunosenescence

**Fig. 3:**
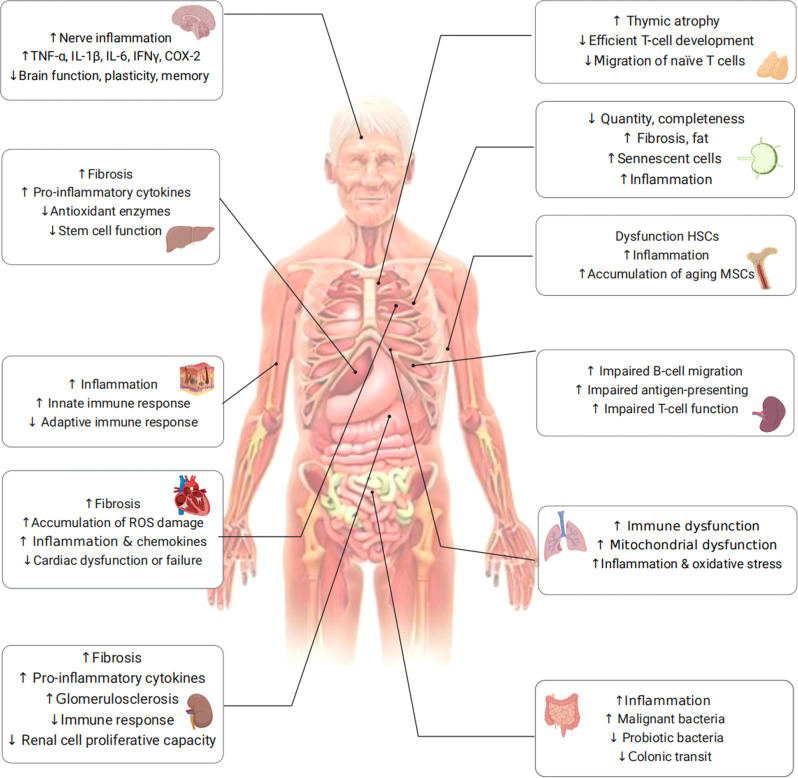
Aging-organ atlas. Aging manifests as a decline in organ function and an increased susceptibility to diseases. Organs are mainly divided into immune organs, sterile organ, and others. Functional changes in cells are shown in each organ

The bone marrow, which serves as the site of hematopoiesis, is a complex environment where bone cells and hematopoietic cells interact with each other. Recent studies have highlighted the importance of the aging bone marrow microenvironment as a key contributor to the aging process. One significant finding is that a higher percentage of senescent bone marrow mesenchymal stem cells (MSCs) have been observed in older individuals compared to younger individuals. This was determined by DNA damage, elevated ROS, and accumulation of SASP-expressing cells. The SASP-generated inflammatory environment can change the expression profile of healthy MSCs and disrupt the expression of factors indispensable for lymphocyte survival (Table 1).^100–102^ Senescent MSCs generating inflammatory factors further impair the function and clonogenicity of young HSCs. Aging has been linked to several hematopoietic system-related issues, such as an increased occurrence of anemias, compromised adaptive immune responses,^103^ and a higher susceptibility to myelodysplastic and myeloproliferative disorders (Fig. 3).^104^

**Table 1:** Senescence-associated secretory phenotype (SASP) list

SASP factors	Secretory profile of senescent cells
*Cytokines*	
IL-1α, -1β, -6, -7, -13, -15, -18; TNF-α	↑
*Chemokines (CXCL, CCL)*	
CCL-17, -20; CXCL-1, -2; IL-8/CXCL-8; IP-10/CXCL-10; Eotaxin-3; GRO-a, -b, -g; HCC-4; MCP-1/CCL2, MCP-2, -4; MIP-1a, -3a	↑
*Growth factors*	
EGF; FGF2; HGF; VEGF; PDGF	↑
*Proteases and regulators*	
BLC; Cathepsin B; COX-1, -2; DPP4; G-CSF; GM-CSF; IFN-γ; M-CSF/CSF-1; MIF; MMP-1, -3, -10, -12, -13, -14; SEMA3F; SERPINs; PAI-1, -2; TIMP-2	↑
*Soluble or shed receptors or ligands*	
ANXA3; Fas; TSPAN8; ICAM-1, -3; OPG; sTNFRI; SGP130; sTNFRII; TRAIL-R3	↑
*Other soluble factors*	
Nitric oxide; PGE2; ROS	↑
*Extracellular matrix (ECM)*	
Collagens; Fibronectin; Laminin; SPARC	↑

SASP is a phenomenon where senescent cells secrete a variety of factors that can have both beneficial and detrimental effects on nearby cells. These factors include cytokines, chemokines, growth factors, extracellular matrix proteins, and proteases, among others. The table shows factors significantly altered between normal and senescent states, arranged by family. Upward arrows indicate an increase in secretion during senescence, crosses indicate no change, and downward arrows indicate a decrease

*BLC* B-lymphocyte chemoattractant, *COX* cyclooxygenase, *DPP4* dipeptidyl peptidase, *ECM* extracellular matrix, *EGF* endothelial growth factor, *FGF2* fibroblast growth factor 2, *G-CSF* granulocyte colony-stimulating factor, *GM-CSF* granulocyte-macrophage colony-stimulating factor, *GRO* growth-related oncogene, *HGF* hepatocyte growth factor, *ICAM* intercellular adhesion molecule, *IGFBP* insulin-like growth factor binding protein, *IL* interleukins, *IP* interferon-gamma-induced protein, *MCP* membrane cofactor protein, *M-CSF* macrophage colony-stimulating factor, *MMP* matrix metalloproteinase, *NGF* nerve growth factor, *OPG* osteoprotegerin, *PAI* plasminogen activator inhibitor; *PDGF* Platelet-derived growth factor, *PGE2* prostaglandin E2, *PIGF* placental growth factor, *ROS* reactive oxygen species, *SCF* stem cell factor, *SDF* stromal cell derived factor, *SEMA3F* semaphorin-3F, *SPARC* secreted protein acidic and rich in cysteine, *sTNFR* soluble tumor necrosis factor receptor, *TIMP* tissue inhibitor of metalloproteinases, *TRAIL* tumor necrosis factor-related apoptosis-inducing ligand, *VEGF* vascular endothelial growth factor

The aging of bone tissue inevitably affects HSCs. With age, red bone marrow is gradually replaced by fat cells, leading to yellow bone marrow formation that inhibits hematopoietic function.^105^ The decreased secretion of nutrient factors by bone marrow stromal cells can result in an enhanced differentiation of HSCs into myeloid cells and a reduced differentiation into lymphocytes. This imbalance in myeloid/lymphoid differentiation is one of the manifestations of HSC aging.^105–107^ Importantly, aged HSCs tend to differentiate more towards myeloid cells, while their ability to support lymphoid cell maturation decreases. This leads to a reduction in the number of precursors for T and B cells with increasing age.^108,109^ Taken together, with aging, the number of HSCs increases, while their function including self-renewal and clonogenicity, decreases. In addition to the HSC changes mentioned above, aging marrow also has decreased Wnt signaling and the accumulation of senescent cells and inflammatory cytokines.^110^

#### Aged thymus promotes T cell-related immunosenescence

The thymus is a central T-lymphatic organ that produces functional initial T-lymphocytes and immune tolerance. In most mammals, aging is accompanied by degeneration of the thymus gland. In humans, thymocyte numbers and hormone secretion levels typically increase during early development and then decrease over time. In addition, the majority of functional cells are substituted with senescent fibroblasts and adipocytes, and stromal cells during thymus aging.^88,111,112^ In the aged mouse thymus, elevated levels of phosphorylated histone H2AX and the p53 binding protein suggest heightened oxidative stress and DNA damage, consequently leading to cellular senescence,^113^ providing support for the notion that the aging thymus exhibits a greater proportion of senescent cells.

Thymic degeneration results in reduced generation of new T-cells, an accumulation of memory T-cells, and a decline in the diversity of T-cell receptors. As a consequence, this leads to a weakened immune response and decreased overall immunity. It has been observed that apparent aging-associated alteration, especially a progressively reduced population of naive T cells, in murine T cell compartment during thymic involution.^114^ However, in human, there is a progressive loss of CD8+ naive T cells while, notably, a relatively stable naive CD4+ compartment is efficiently maintained via homeostatic proliferation.^115–117^

Consequently, both in mice and humans, age-related variations in the production of naive T cells from the thymus result in qualitative disparities in the overall T cell repertoire.^118–121^ In addition, it has been observed that naive T cells from older individuals exhibit reduced responsiveness to the superantigen toxic shock syndrome toxin-1 compared to younger individuals,^122,123^ which may be caused by low dual specificity phosphatase 6 levels in naive T cells, resulting in a rise in the threshold of TCR activation.

#### Aged spleen promotes T and B cell-related immunosenescence

The spleen acts as a secondary lymphoid organ promoting immune defense and is the main pivotal organ for initiating the activities required for the adaptive immune responses. During the aging process, significant alterations occur in the cellular composition and microarchitecture of the spleen. The clear distinction between T-cell and B-cell areas within the white pulp becomes less defined, and there are noticeable changes in the organization and functionality of marginal zone macrophages, stromal cells, and marginal metallophilic macrophages.^124,125^

Furthermore, recent advancements in single-cell RNA sequencing studies have revealed that the proportion of T cells in the spleen decreases with age, while the relative abundance of plasma cells increases.^126^ Impaired migration of B cells and the phagocytic capacity of macrophages in the marginal zone can also be seen in aged spleens.^127^ Also, impaired function of microenvironment-mediated antigen-presenting cells was observed, which may provide an explanation for the observed delayed responses to stimulation even in T cells derived from young HSCs (Fig. 3).^102,128,129^

#### Aged lymph nodes promote immunosenescence

Lymph nodes serve as crucial sites where T cells and B cells reside and where immune responses are initiated, playing a vital role in establishing an effective immune response. However, the number, integrity, and functionality of lymph nodes undergo significant declines with age, as evidenced by previous studies.^102,125^ The exact cause of lymph node atrophy remains unknown; however, it can result in the deterioration of the microenvironment where immune cells reside, thereby negatively impacting immune function. Age-related alterations in cellularity and the functionality of different cell types within the lymph nodes have been extensively documented.^130^ Specifically, the number of fibroblastic reticular cells in lymph nodes diminishes, resulting in a compressed and less reticular stromal network.^131^ In addition, older individuals, aged 60 years and above, exhibit increased fat deposition and fibrosis in their lymph nodes.^124,125^ Moreover, stromal cells within aged lymph nodes exhibit reduced replication potential when stimulated and are unable to maintain a balance of naive T cells.^126,127,131^ The accumulation of senescent cells in lymph nodes, along with heightened inflammation, may negatively impact the migration and recruitment of immune cells, thereby serving as detrimental factors (Fig. 3).^130^

### Sterile organs

#### Brain

The main cause of brain aging appears to be neuroinflammation^132^ via aged brain cells and a weakened immune system. The process of brain aging significantly contributes to the decline of various cognitive functions, encompassing decreased speed of information processing, reduced capacity of working memory, impaired spatial memory, and diminished plasticity (Fig. 3).^133^

Aging of brain cells including neurons and glial cells (i.e., microglia and astrocytes) leads to the upregulation of inflammatory-related pathways, causing brain function weakness and increased inflammation damage. During the aging process, microglia gradually lose their ability to efficiently clear misfolded proteins that are linked to neurodegeneration. This impairment in protein clearance significantly contributes to the neuroinflammatory response observed in the brain, with microglia playing a central role in this process. Except for the supporting role, Shao et al. found that astrocytes can also be a mastermind of neuroinflammation, depending on the Dopamine D2 receptor (Drd2), normally an important brake on it.^134^ During aging, the level of Drd2 and its ligand dopamine both decline with high neuroinflammation in the brain. Subsequently, activated astrocytes produce SASP factors, such as IL-1β, IL-6, TNF-α, IFN-γ, COX-2, and other inflammatory factors (Table 1). In turn, these factors further promote astrocyte activation. The excessive production of pro-inflammatory mediators disrupts the intricate equilibrium necessary for the induction of long-term potentiation, leading to a decrease in the production of brain plasticity-related molecules such as BDNF and IGF-1, consequently impairing synaptic plasticity.^135^ Remarkably, even older adults without neurological impairments demonstrate a gradual escalation in neuroinflammation, characterized by elevated homeostatic levels of inflammatory cytokines and reduced production of anti-inflammatory molecules (Fig. 3).^136^

Immunosenescence and inflammaging can both contribute to neuroinflammation, resulting in impaired neuronal function and the accumulation of brain tissue damage.^137,138^ Consequently, various central nervous system disorders, including Alzheimer’s disease, Parkinson’s disease, and stroke, are characterized by degenerative neurological conditions.^139^

#### Heart

Most cardiac tissue is composed of cardiomyocytes, cardiac fibroblasts, and macrophages. The aging process in the heart is characterized by the gradual occurrence of several hallmarks, including progressive cardiomyocyte hypertrophy, the gradual onset of cardiac fibrosis, and the presence of inflammation (Fig. 3).^140^

Hypertrophic cardiomyocytes, characterized by heightened oxygen and energy requirements, create a hypoxic environment of low oxygen levels. This imbalance in oxygen levels leads to the generation of excessive free radicals, which can potentially damage cellular components. In response to hypoxia, cardiomyocytes release pro-inflammatory cytokines and chemokines. These molecules stimulate an immune response and contribute to an increase in the number of macrophages within the left ventricle.^140^ In addition, because mature cardiomyocytes have a low rate of proliferation, the injured area in the aging heart is replaced by fibrotic scar tissue, resulting in organ failure.^141^

The main effector cells in cardiac fibrosis are activated myofibroblasts. Long-term inflammation promotes cardiac and vascular fibrosis. The cardioprotective effects of AMPK and GDF11 on cardiomyocytes have been extensively documented, and the decline in AMPK and GDF11 expression associated with aging is likely a contributing factor to the heightened cardiac fibrosis observed during the aging process.^142^

In the steady-state heart, macrophages play a crucial role in eliminating senescent and dying cells, contributing to the normal homeostatic maintenance of the myocardium and facilitating tissue repair following injury. However, in the aging heart, macrophages recruited to the site of infarction exhibit a pro-inflammatory M1 phenotype initially, but subsequently transition to an anti-inflammatory M2 phenotype after myocardial infarction (MI). This phenotypic switch promotes angiogenesis and scar formation, aiding in the recovery process.^143^

In addition, vascular smooth muscle cells (VSMCs) play a crucial role in coordinating vascular function alongside endothelial cells, regulating blood pressure, vascular tone, and blood flow. However, during the aging process, the dysfunction and decline of VSMCs have a detrimental impact on the structural integrity of the aorta, ultimately leading to the development of transthoracic aortic aneurysms.^142^ Classical molecular IGF-1 signaling causes cardiac hypertrophy and heart failure.^142^ IGF-1 increases cellular senescence in VSMCs by inducing DNA damage and increasing ROS production via the p53 pathway.

#### Kidney

As individuals age, the kidneys undergo various structural impairments, such as fibrosis, and experience functional issues, including mitochondrial dysfunction (Fig. 3).^144–146^ Moreover, older individuals become more susceptible to acute kidney injury (AKI) and chronic kidney disease (CKD).^123,147^ Age-related alterations in multiple cell types, such as tubular epithelial cells, resident and circulating leukocytes, contribute to kidney injury. Proximal tubular cells depend on autophagy to effectively eliminate defective mitochondria and other organelles under both normal and pathological conditions.^148,149^ However, during aging, their diminished proliferative capacity leads to impaired clearance abilities.^150^

Aging in the kidneys is associated with various physiological changes, including chronic low-grade inflammation. This inflammatory state, often referred to as inflammaging, has been observed to have detrimental effects on the kidneys. Chronic low-grade inflammation in the kidneys can impair the normal repair mechanisms that occur following injury. This inflammation hampers intrinsic cellular repair mechanisms following injury and promotes immunosenescence and organ damage.^151,152^ Elderly individuals exhibit immunological phenotypes characterized by reduced numbers of naive lymphocytes, increased pro-inflammatory T cells, and diminished phagocytic activity in monocyte lineage cells, similar to CKD patients. These alterations in the kidneys form the basis for prevalent pathological conditions that are commonly observed in both elderly individuals and patients with CKD.^123^

#### Liver

Aging raises the risk of chronic liver disease and liver fibrosis, which is highly related to hepatic stellate cells, hepatocytes, and macrophages. Liver cells initially activate compensatory mechanisms in response to time-dependent damage caused by aging, which can lead to the development of pathologies of the liver if overstimulated.^153^ Activated hepatic stellate cells are the major functional population during liver fibrogenesis.^154^ During senescence, their replication, immune-recruiting signals, and clearance are important for the regulation of liver fibrogenesis.^155^ An illustration of the contribution of senescent hepatocytes to hepatic stellate cell activation and liver fibrogenesis is evident in p53-deficient mice with nutrition-induced steatohepatitis. It was discovered that these mice displayed reduced levels of hepatocyte p21, as well as decreased activation of hepatic stellate cells and expression of fibrotic markers such as SMA and collagen.^156^ This finding supports the involvement of senescent hepatocytes in the activation of hepatic stellate cells and the development of liver fibrosis. Furthermore, M2 macrophages secrete pro-fibrogenic mediators, including TGF-β1, which promote the progression of liver fibrosis.^157^ In brief, the recruitment and mobilization of immune cells, the accumulation of inflammation, and the activation of hepatic stellate cells and hepatocytes contributes to the development of liver fibrosis and the aging process (Fig. 3).

### Other organs

#### Skin

During the aging process, the skin accumulates senescent cells that, despite their inability to divide, remain metabolically active. These senescent cells exhibit an altered secretome known as SASP, which significantly disrupts the skin microenvironment.^158^ For instance, senescent dermal fibroblasts secrete a higher amount of extracellular vesicles (EV) compared to their non-senescent counterparts. This increased EV secretion hampers the normal differentiation of keratin-forming cells and compromises the skin’s barrier function. In addition, it triggers the elevated production of the pro-inflammatory cytokine IL-6.^159^

Furthermore, skin aging can occur due to age-related factors or exposure to environmental stressors like ultraviolet radiation.

The process of skin aging can also have systemic effects on the overall aging process of the body, primarily through the activation of SASP.^160^ The presence of p16-positive cells in the skin, which is a marker of cellular senescence, has been found to be associated with markers of CD4+ T-cell senescence and biological age.^161,162^ Notably, the microbiome of skin has been found to predict a person’s actual age accurately.^160,163^ While the numbers of CD4 T cells remain consistent with age, the levels of CD8 T cells are higher in older skin compared to younger skin.^164^ The ratio of cutaneous CD4 T cells to CD8 T cells is greater in aged individuals, but the number of CD4 T cells is not elevated in aged skin.^165^ Moreover, aged skin exhibits increased numbers of regulatory T cells (Tregs)^166^ and elevated expression of the immunosuppressive receptor PD-1,^165,167^ which may contribute to weakened adaptive immunity. These changes could be a response to an inflammatory state exacerbated by impaired epidermal barrier function or fibroblast senescence, further promoting an inflammatory microenvironment (Fig. 3).

#### Lung

The aging process brings about notable transformations in the structure and function of the lungs, including a decline in mucociliary clearance and heightened vulnerability to pulmonary infections.^168,169^ These alterations contribute to the onset and progression of various lung diseases like idiopathic pulmonary fibrosis (IPF) and chronic obstructive pulmonary disease (COPD).^169^ Several cell types within the lungs undergo modifications during aging, including respiratory epithelial cells, lung progenitor cells, lung immune cells, and lung interstitial cells.^169^ Among these cell types, alveolar epithelial type II cells (AT2) are a significant population responsible for regenerating the alveolar parenchyma. However, as these cells age, the airway epithelium experiences quantitative and qualitative defects. The number of basal and spherical cells decreases, while the count of AT2 cells remains unchanged but exhibits impairments in self-renewal and differentiation capacity.^170–172^ Moreover, age-related changes in the lung environment, such as alterations in extracellular matrix (ECM) components, tissue and circulating cytokines, SASP, and structural abnormalities, can lead to abnormal intercellular communication mechanisms. This is evident through distorted interactions with microbial pathogens and a shift in innate and adaptive immunity towards increased inflammation, disrupted adaptive immune responses, and impaired immune surveillance (Fig. 3).^173^

The increased susceptibility of elderly individuals to lung diseases can be ascribed to age-related changes in immunity and anti-infection responses. The phagocytic capacity of pulmonary and alveolar macrophages diminishes with age, impairing the clearance of pathogens from the lungs.^51,174,175^ Dendritic cells, neutrophils, and NK cells also experience age-related alterations in their numbers and functionality.^169^ In addition, the aging process is linked with a decrease in CD4, and CD8 T cell populations. The decline in naive T cell numbers is accompanied by an increase in the number of memory T cells. The CD4 to CD8 lymphocyte ratio in bronchoalveolar lavage fluid tends to rise with age, indicating a reduction in the pool of naive T cells available for conversion into memory cells in response to new antigens. Furthermore, aging is associated with reduced CD4 and CD8 T cell responses, diminished TCR repertoire diversity, impaired Th cell differentiation, and reduced Th cell activity.^176^ These age-related changes in T cell number and function can compromise influenza vaccination immunity and cytotoxicity against the virus. The adaptive immune response to antigens also declines with age, which explains why older individuals are more susceptible to environmental stimuli. Notably, immune cell disorganization associated with aging may contribute to the heightened severity of COVID-19 and chronic obstructive pulmonary disease (COPD) in the elderly.^169,177^

To recapitulate, age-related changes in T cell-mediated adaptive immune responses enhance vulnerability to infectious agents and result in severe diseases.

#### Gut

Age-related perturbations in the gut microbiome have emerged as crucial factors contributing to age-related pathological conditions, including chronic inflammation,^178^ neurodegeneration,^179^ cognitive decline,^180^ and type 1 and type 2 diabetes.^181^ The gut microbiota comprises probiotic, commensal, and pathogenic bacteria, and the imbalance between intestinal flora and aging mutually influences and exacerbates each other. Older adults (>65 years) exhibit reduced microbiota diversity compared to adults, along with greater inter-individual variation in microbiota composition.^182^ This is characterized by diminished populations of beneficial bacteria such as Bifidobacterium, Bacillus, *E. coli*, Clostridium XIV, Blautia coccoides-Eubacterium rectal, and Bacteroidetes, and increased presence of Enterobacteriaceae.^183^ However, it’s important to note that data regarding age-related changes in microbiome composition can vary among populations.

Age-related alterations in the intestinal microbiota, particularly due to prolonged immune system stimulation, can contribute to the accumulation of inflammation and a decline in immune system function known as immunosenescence.^184^ Interestingly, two previous studies have indicated that changes in the relative abundance of gut microbiota are more likely to be influenced by inflammation rather than age, with TNF playing a significant role.^185,186^ This suggests that the changes in gut microbiota are more closely related with inflammatory processes rather than solely being a consequence of aging (Fig. 3).^187,188^

## Mechanisms of inflammaging

### Consensus features of inflammaging

While the precise interpretation of senescent cell markers remains incomplete and requires further investigation, there is a consensus regarding certain essential characteristics of senescent cells, primarily focusing on the SASP (Table 1). The SASP, considered to be molecular inflammation, is a universal, dynamic, and complex phenomenon arising with cellular senescence. It is the phenomenon of senescent cells secreting pro-inflammatory cytokines.^189^ The SASP possesses the capacity to perpetuate senescence itself or influence the surrounding tissue microenvironment, consequently affecting the entire organism. Classic SASP factors contain pro-inflammatory and immune-modulatory cytokines, chemokines, proteases, and growth factors (Table 1).^190–193^ According to its complex composition, the SASP has been implicated in the majority of the nonautonomous effects observed in senescent cells, including inflammation, immune evasion, tumor promotion, senescence reinforcement, paracrine senescence, and so on.^194^ Recent analysis has identified a set of shared components within the SASP that are consistent across various inducers of senescence and different cell types. Interestingly, some of these components overlap with aging markers observed in human plasma, including serine protease inhibitors, stanniocalcin 1, and growth differentiation factor 15.^190^ Furthermore, dysfunctional mitochondria,^195^ persistent DNA damage response,^196^ CCAAT/enhancer-binding protein β (C/EBPβ) and NF-κB,^197^ mTOR, and other factors are involved in regulating SASP.^194^

In addition, SASP encompasses additional characteristics such as lipofuscin accumulation within lysosomes, increased cytoplasmic DNA, activation of anti-apoptotic pathways, and alterations in the nucleus, including the loss of Lamin B1, telomere shortening, senescence-associated heterogeneous chromatin aggregation, and the presence of telomerase-associated foci.^198^ At the transcriptional level, p16 and p21 are the most commonly used markers to identify senescent cells. These features and markers have been extensively employed to detect senescent cells in various tissues, both in the context of individual senescence and other pathological conditions.^199^

### Triggers of inflammaging

Inflammaging develops from cold-inflammaging with a less than 2-fold increase of pro-inflammatory mediators in plasma, compared to healthy adults.^200^ This slightly altered level is a positive response for maintaining homeostatic stability. However, during aging, the homeostasis imbalance arises and progresses, leading to increased cytokine response (2- to 4-fold increase) mediated by the chronic activated innate immune system. The transition is highly influenced by several vital triggers, including cellular senescence with the secretion of SASP, which have been already mentioned above, dysbiosis caused by microbiome and their metabolites, and endogenous molecular garbage caused by abnormal cell death.

#### Oxidative stress

Oxidative stress leads to oxidative damage to biomolecules (especially DNA),^201^ causing endogenous damage-associated molecular patterns (DAMPs) production and cytokine release in the organism.^202,203^ Cytokines activate downstream signaling pathways of pattern recognition receptors,^203^ causing systemic chronic inflammatory responses in the body.^204^ Consequently, oxidative stress is recognized as a concurrent occurrence within the inflammatory process, amplifying the inflammatory response through oxidation. At the same time, inflammation promotes oxidation through inflammatory mediators.^205,206^ Based on the close relationship among oxidative stress, inflammation, and aging, Dela Fuente et al. proposed the theory of aging by oxidation-inflammation (oxi-inflamm-aging)^207,208^ and concluded that oxidative stress leads to inflammatory aging. Oxidative stress has been ensured as a crucial factor for cellular senescence through shortening telomere and causing DNA double-strand breaks.^209,210^ Moreover, infections,^211^ environmental pollution,^212,213^ and adverse lifestyle habits^214^ can increase oxidative stress.

#### Microbiome

Recently, there has been an increasing focus on studying age-specific changes in the intestinal microbiome and its role in regulating inflammation. A healthy gut microbiome is essential for body metabolism, infection resistance, inflammation regulation, prevention of autoimmunity and cancer, and brain-gut axis regulation.^215^ However, with age, there is a decrease in beneficial microorganisms^216,217^ and an accumulation of potentially pro-inflammatory microorganisms in the gut,^218^ leading to a change in microbial composition and a decrease in microbial diversity. Moreover, this phenomenon exists simultaneously in species such as Drosophila,^219^ fish,^220^ mice,^221^ rats,^222^ and humans.^223^ The detailed gut microbiota changes with aging have been collected and discussed by Du et al.^224^ Recent studies have revealed that the transplantation of fecal matter from young donors into the gastrointestinal tract of middle-aged fish can effectively prolong lifespan and delay the onset of behavioral decline,^220^ and that fecal transplantation from young mice slows HSC senescence in the bone marrow.^225^ Lachnospiraceae and tryptophan-associated metabolites have emerged as key players in important biological processes, but the exact mechanism and involvement of other factors are not yet clear. Nonetheless, it is evident that the senescence-related remodeling of microorganisms mediates the accumulation of chronic inflammation, which is highly correlated with their metabolites and their induced immune responses.

### Inflammatory cell death

Eukaryotic cells possess the ability to activate various self-destructive mechanisms, but the type of cell death can be classified as either inflammatory or non-inflammatory. In normal tissues, cell death serves as a highly conserved process that promotes a stable cell population through the elimination of surplus, impaired, or aged cells. Consequently, the human body generates over 150 billion deceased cells on a daily basis.^226^ A newly developed conception, garb-aging, reveals that the production of inflammatory cell death modalities, endogenous molecular garbage (e.g., mitochondrial RNA, misplaced molecules, and cell debris), is a causal inflammatory stimuli that can accelerate inflammaging.^227,228^ Certain cytokines, such as IL-1β and IL-6, have clearly emerged as key to promoting inflammaging.^83,229–232^ Moreover, inflammatory death of internal cells due to exogenous factors such as infection also promotes the progression of inflammaging.^17,233^ During aging, the imbalance between the increased production and decreased disposal via autophagy, mitophagy, and proteasome, stimulus the innate immune system and thereby triggers the body from a pre-inflammatory state towards a pro-inflammatory state.^234^

#### Necrosis

As the body ages, tissues and cells gradually experience damage, leading to a decline in their abilities and functions. Aging cells may be more susceptible to damage from external stimuli, increasing the risk of necrosis. In addition, certain age-related diseases such as cardiovascular diseases and neurodegenerative disorders may be accompanied by cellular necrosis.

**Fig. 4:**
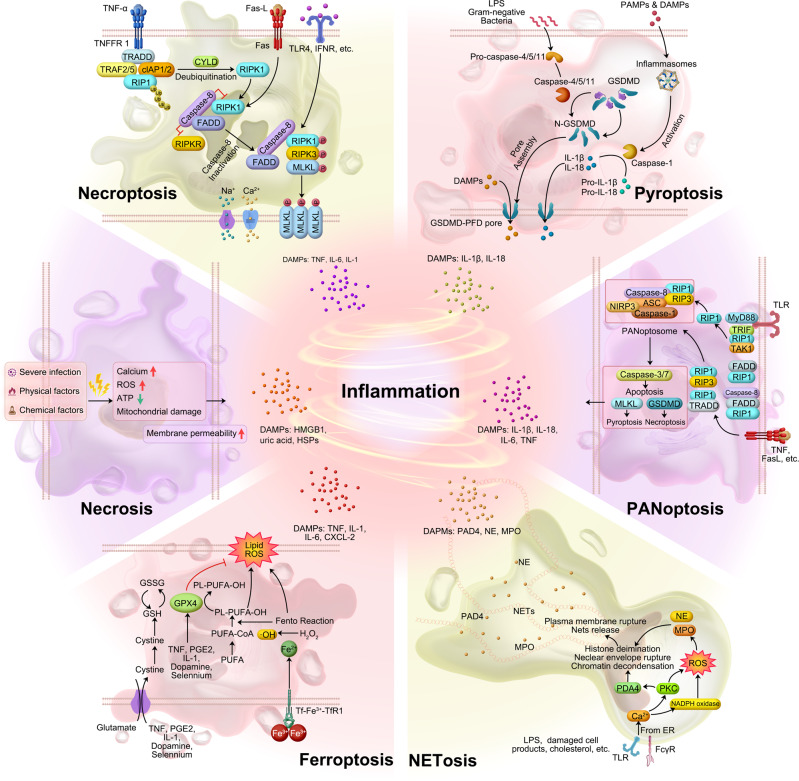
Schematic diagram of six inflammatory cell death molecular patterns. The source of inflammation comes from cell death in addition to the release of SASP from senescent cells. Both immune response-mediated and damage signaling-mediated cell death promote inflammation to some extent. Various modes of cell death (in addition to apoptosis) release large amounts of inflammatory factors

Necrosis is traditionally considered an unprogrammed and unregulated form of cell death that occurs due to overwhelming external stimuli.^235^ It is characterized by cellular swelling, loss of membrane integrity, release of intracellular contents (DAMPs and pathogen-associated molecular pattern, PAMPs) into the extracellular environment, an increase in intracellular calcium concentration, and the generation of ROS. These events ultimately lead to irreversible cellular damage. DAMPs, such as HMGB1, uric acid, nucleosomes, and members of the heat shock protein family (HSP 70, HSP 60, and GP96), can directly or indirectly activate and recruit immune cells, thereby triggering inflammation or immunosuppression.^236^ It is important to note that these factors can be released during the entire process of cell death, even when cells are still metabolically active. Consequently, cells in the process of dying may contribute to carcinogenesis even before the appearance of obvious necrotic changes (Fig. 4).^236^

#### Necroptosis

Necroptosis (previously named programmed necrosis) is a regulated form of inflammatory necrosis that occurs when apoptosis (the programmed cell death process) fails. Its identification questioned the conventional notion that necrosis is exclusively an inert process induced by overwhelming stress. Necroptosis is distinguished by the early disruption of plasma membrane integrity, release of intracellular contents, and enlargement of organelles. Apoptosis is generally regarded as non-immunogenic since the regulated dismantling of apoptotic cells restricts the liberation of DAMPs. However, necroptosis triggers inflammation through the massive release of DAMPs from the disintegrating cell.^237^

The contribution of DAMPs from dying cells in the RIPK1-RIPK3 inflammasome-dependent pathway of cytokine production varies. Upregulation of RIPK3 has been observed in hepatocytes, suggesting that RIPK3-dependent necroptosis may play a role in inflammation and hepatocyte death. Immunostaining with antibodies recognizing phosphorylated mixed lineage kinase domain-like protein (MLKL) serves as a specific marker of necroptosis.^238,239^ In addition, immunostaining using antibodies that target phosphorylated MLKL has been identified as a specific marker for necroptosis.^240^

Focus on liver, its aging has been linked to an increase in necroptosis, and this process has been found to contribute to chronic liver inflammation, which in turn appears to be involved in the development of liver fibrosis.^241^ On the other hand, in the livers of old mice (specifically, those aged 18 months and older), there was a significant upregulation of phosphorylated MLKL and MLKL oligomers, which are markers associated with necroptosis. In addition, the phosphorylation of RIPK3 and RIPK1, two key proteins involved in necroptosis signaling, was also significantly increased in the livers of old mice compared to young mice. In comparison to young mice, hepatocytes and liver macrophages from old mice had higher levels of necroptosis markers and higher expression of pro-inflammatory cytokines M1 macrophage markers, pro-inflammatory cytokines (TNF-α, IL-1, and IL-6), and fibrosis markers. In the livers of old mice, short-term treatment with the necroptosis inhibitor necrostatin-1s (Nec-1s) reduced necroptosis, M1 macrophage markers, cellular senescence, fibrosis, and pro-inflammatory cytokines.^241^ Importantly, nerve injury-induced protein 1 (Ninjurin1/Ninj 1) plays a crucial role in facilitating the ultimate breach of the plasma membrane that takes place in necroptosis, pyroptosis, and secondary necrosis. Secondary necrosis refers to the phenomenon where cells undergoing apoptosis fail to be engulfed by adjacent phagocytes (Fig. 4).^242^ These findings suggest an age-associated dysregulation of necroptosis signaling, indicating a potential role for necroptosis in aging and related pathologies.

#### Pyroptosis

As the body ages, tissues and cells gradually experience damage, leading to a decline in their abilities and functions. Similar to other forms of inflammatory cell death, aging cells may also be more susceptible to damage from external stimuli that can trigger pyroptosis, increasing the risk of pyroptosis occurrence. In addition, age-related diseases such as neurodegenerative disorders and cardiovascular diseases may be accompanied by cellular pyroptosis. Specifically, pyroptosis may play a specific role in aging. Inflammation and cell death have important regulatory roles in aging, and pyroptosis, as an inflammatory form of cell death, may contribute to the inflammatory response and cellular dysregulation in the aging process.^243^ Moreover, pyroptosis may be involved in the development and progression of age-related diseases.

In the non-classical pathway, human-derived caspase-4, 5, and murine-derived caspase-11 can be activated upon direct contact with bacterial lipopolysaccharide (LPS). LPS cleaves gasdermin D (GSDMD), which indirectly activates caspase-1, leading to pyroptosis. Alternatively, caspase-1 can be recruited and activated by inflammatory vesicles that detect danger signals. Activated caspase-1 cleaves and activates inflammatory factors, which in turn cleave the N-terminal sequence of GSDMD. This results in the binding of GSDMD to the membrane and the generation of membrane pores, ultimately leading to pyroptosis (Fig. 4). Evidence of pyroptosis, coincided with elevated levels of IL-1 and IL-18, and inflammasome activation, has been illustrated in various conditions such as atherosclerosis, neurodegenerative diseases, cancer, and chimeric antigen receptor (CAR)-T therapy.^244–247^

#### Ferroptosis

As the body ages, changes in iron levels and iron metabolism may occur. The accumulation of iron in cells during the aging process may be associated with the development of age-related diseases such as neurodegenerative diseases^248^ and cardiovascular diseases.^249^ This iron accumulation can lead to increased generation of ROS within cells, thereby triggering iron-dependent cell death known as ferroptosis. The main drivers of ferroptosis are the inactivation of the lipid repair enzyme glutathione peroxidase 4 (GPX4) and the induction of ROS, particularly lipid ROS. GPX4 plays a cytoprotective role by reducing cellular lipid hydroperoxide levels, which are associated with inflammation. In cancer cells, certain inflammatory cytokines such as TNF, PGE2, IL-1, and IL-6 have been shown to directly affect GPX4 levels and activity. Treatment with TNF, for example, downregulates GPX4, leading to ferroptosis.

Ferroptosis is an inflammatory form of cell death that is distinct from apoptosis. It is characterized by iron-dependent lipid peroxidation and can contribute to various pathological processes, including neurodegenerative diseases, inflammatory diseases, autoimmune diseases, and cancer. The inactivation of the lipid repair enzyme glutathione peroxidase 4 (GPX4) and the induction of ROS, particularly lipid ROS, are the main causes of iron death. GPX4 has been shown to have a cytoprotective effect by lowering the levels of cellular lipid hydroperoxides.^250^ Several pro-inflammatory cytokines, including TNF, PGE2, IL-1, and IL-6, have been demonstrated to exert a direct influence on the levels and function of GPX4 within cancer cells;^251^ for example, TNF treatment causes GPX4 downregulation that can lead to ferroptosis.^252^ High mobility group box 1 (HMGB1), a DAMP, has been implicated in inflammation and its pathogenesis.^253,254^ In the context of ferroptosis, inhibiting HMGB1 release has been shown to limit the inflammatory response during cell death. Anti-HMGB1 antibodies have demonstrated their ability to reduce the inflammatory response in macrophages induced by ferroptotic cells.^252^ Ferroptosis inhibitors have shown promise in the treatment of certain diseases due to their anti-inflammatory properties. In an oxalate-induced mouse model of AKI, evidence of inflammation was observed, and the ferroptosis inhibitor Ferrostatin-1 successfully inhibited neutrophil infiltration and the expression of pro-inflammatory cytokines such as CXCL-2 and IL-6.^255,256^ Conversely, the ferroptosis inducer RSL-3 significantly increased the protein levels of pro-inflammatory cytokines like TNF, IL-1, and IL-6, exacerbating hepatosteatosis, lobular inflammation, and apoptosis (Fig. 4).^257^ The results indicate a possible interplay between ferroptosis and inflammation.

In addition, certain biological processes and molecular mechanisms associated with aging may be related to ferroptosis. During the aging process, alterations in cellular function and metabolism can increase the sensitivity of cells to external stimuli, including sensitivity to ferroptosis. Recent studies have reported the involvement of ferroptosis as a mechanism that promotes skeletal muscle aging.^258^ With skeletal muscle aging, there is a decreased expression of Tfr1 and an increased expression of Slc39a14, which is enriched on the cell membrane surface of aging mouse skeletal muscle cells. This increase in Slc39a14 leads to enhanced non-transferrin-bound iron uptake, resulting in the accumulation of free iron ions within skeletal muscle and the occurrence of ferroptosis.^258^

Lastly, the interaction between ferroptosis and aging may be bidirectional. On one hand, ferroptosis may play a role in the development of certain age-related diseases, accelerating tissue and cellular aging processes. On the other hand, the cellular functional and metabolic changes that occur during the aging process may increase the sensitivity of cells to ferroptosis, further promoting disease progression.

#### NETosis

NETosis is a special form of cell death closely associated with inflammation and immune response. It is a cell death program executed by neutrophils and is characterized by the release of net-like structures (neutrophil extracellular traps/ NETs). These structures are composed of DNA, histones, and microbial toxins, among other components, and serve the purpose of capturing and killing microorganisms.^228^ NETosis plays a significant role in various pathologies, including COVID-19, Kawasaki syndrome, and rheumatoid arthritis (RA). Excessive NETosis has been implicated in the development of cytokine storms and thrombosis.^259^ In COVID-19, NETosis can be caused by virus-infected epithelial and endothelial cells, thereby activating inflammatory cytokines and platelets. Excessive NETosis, accompanied by increased circulating free DNA and Neutrophil Extracellular (NE)-DNA complexes, is also found in acute Kawasaki syndrome, a vasculitis occurring in children.^260^

In RA, the disease pathology is characterized by the accumulation of DNA-MPO complexes and the presence of antibodies targeting guanylated histones (NETosis markers).^261,262^ In myocarditis, NETosis probably promotes PMN trafficking via MK and substantially contributes to cardiac inflammation.^263^ In systemic lupus erythematosus, NETosis activates the plasmacytoid and induces the production of IFN-α and ROS, which contribute the following further inflammation.^264–267^ On the other hand, the anti-microbial effects of NETosis have been observed to slow down the spread of pathogens in infected lesions. NETs in staphylococcal skin infections inhibit the penetration of pathogens into the bloodstream.^268^ Knockout of the PAD4 gene in mice prevents NET formation and leads to more severe necrotizing fasciitis caused by streptococcus pyogenes. In summary, NETosis could cause inflammation or conversely slow the onset of age-related diseases (Fig. 4).

The ability of neutrophils to undergo NETosis may be affected by aging.^269^ Senescent neutrophils exhibit several distinct characteristics during NETosis, including a reduced capacity to release NETs, instability in the quality of formed NETs, and decreased activity of DNA degrading enzymes (DNases) within NETs. These age-related changes can result in impaired functionality of aging neutrophils, leading to deficiencies in their ability to effectively combat microbial infections and regulate inflammatory responses.

Moreover, aging is often accompanied by a phenomenon called inflammaging, which refers to a chronic low-grade inflammatory state. Inflammaging can further contribute to the occurrence of NETosis. This persistent inflammatory condition enhances the activity of inflammatory cells, including neutrophils, thereby increasing the likelihood of NETosis. A previous study found that aged mice exhibited an increased propensity for NETosis compared to younger mice. This heightened NETosis activity was associated with the activation of peptidylarginine deiminase 4 (PAD4), an enzyme involved in the formation of NETs. The excessive formation of NETs, in turn, was implicated in the development of age-related organ fibrosis.^270^

In conclusion, the process of aging can adversely affect the ability of neutrophils to undergo NETosis. Senescent neutrophils may experience limitations in NET release, compromised stability of formed NETs, and reduced DNase activity within NETs. In addition, the presence of inflammaging, the age-associated inflammatory state, can intensify the occurrence of NETosis by stimulating inflammatory cell activity. However, further research is necessary to fully comprehend the intricate mechanisms and interactions between aging and NETosis.

#### PANoptosis

PANoptosis, is a united modality of inflammatory programmed cell death, accompanied by markers of apoptosis, necrosis, and pyroptosis pathways.^271–275^ Influenza A virus was first discovered to cause PANoptosis, followed by many other infections, of bacterial, fungal, and viral origin.^275^

PANoptosis is involved in the occurrence of cytokine storms (CS) characterized by excessive cytokine production.^276^ The combination of TNF-α and IFN-γ activates the JAK/STAT1/IRF1 signaling pathway, leading to the production of nitric oxide (NO). This NO release triggers PANoptosis through the involvement of GSDME (pyroptotic), CASP8/3/7 (apoptotic), and pMLKL (necroptotic) pathways. In vivo, blocking CS by giving mice anti-TNF-α and anti-IFN-γ antibodies prevents death from SARS-CoV-2 infection, hemophagocytic lymph histiocytosis, and LPS shock (sepsis).^276^ This highlights the critical role of TNF-α and IFN-γ released from PANoptosis in driving cytokine storms during infections and inflammatory conditions.^275^ Cytokines or PAMPs trigger the assembly of a multiprotein complex called the PANoptosome. This complex includes various molecules necessary for the activation of downstream programmed cell death (PCD) effectors such as GSDMD, GSDME, CASP3/7, and MLKL (Fig. 4). No direct evidence yet links PANoptosis to aging. However, aging can affect cell responses to inflammation and cell death. Further research is needed to explore the potential connection between aging and PANoptosis, shedding light on its impact on immune and cell death mechanisms.

In summary, DAMPs from senescent, damaged, and dying cells trigger various cell death modalities, including necrosis, pyroptosis, necroptosis, PANoptosis, NETosis, and ferroptosis (Fig. 4). DAMPs bind to specific receptors, initiating inflammation and orchestrating a coordinated response involving immune cells. This response includes the recruitment of neutrophils and monocytes, which play crucial roles in tissue repair and healing processes. When leukocytes fail to clear immunostimulatory molecules, inflammation persists, which further causes cancer and aging. Endogenous DAMPs can activate PRRs and non-PRR transmembrane proteins, resulting in massive inflammation, cellular senescence, diseases of the organs, and aging.^277–279^ The shift of understanding in aging mechanisms, from the cellular and organ level to the molecular level, aid in identifying novel targets for anti-inflammatory therapies and effective anti-aging interventions.

## Classical models to study aging

Aging model systems can simulate human physiological and pathological processes to reveal aging mechanisms and guide anti-aging research. To date, aging models consist of in vitro models (e.g., physical, chemical, and biological induced models) and in vivo models (e.g., animal models, premature aging models, and centenarian).

### In vitro models

Here, we describe the main in vitro models of senescence used in research, classified according to different stimuli: replicative senescence (RS), oncogene-induced senescence (OIS), and chemotherapy-induced senescence (CIS).

#### Replicative senescence (RS) models

Replicative senescence is closely associated with the shortening of telomeres. In the laboratory aging of human diploid fibroblasts (HDFs), as the cells undergo a certain number of population doublings, telomeres become shorter, leading to cell cycle arrest, reduced cell saturation density, and increased cell surface and volume.^280^ Hydrogen peroxide is commonly used to induce stress-induced premature senescence (SIPS), which shares similarities with replicative senescence. When young HDFs are exposed to prolonged low doses of hydrogen peroxide, they enter irreversible G1 cell cycle arrest and exhibit senescence-associated beta-galactosidase activity. These cellular senescence markers are accompanied by increased expression of p21, gadd45, and enhanced p53 binding activity.^281^ In addition, DNA repair capability decreases, and telomere shortening accelerates. Hydrogen peroxide-induced senescence also triggers inflammation, characterized by the upregulation of pro-inflammatory cytokines such as IL-6, TNF-α, and MCP-1.^282,283^

#### Oncogene-induced senescence (OIS)

Oncogene-induced senescence (OIS) is observed following the activation of various oncogenes such as B-RAFV600E or H-RAS G12V, as well as the loss of tumor suppressor proteins like PTEN or NF-1, in different cell types.^284^ OIS is often associated with DNA replication stress and hyper-replication. It is characterized by the upregulation of CDK inhibitors, including p15INK4B, p16INK4A, p21CIP1, and an increased senescence-associated β-galactosidase (SA-β-Gal) activity.^285,286^

Kuilman et al. discovered that OIS is specifically associated with the induction of an inflammatory gene expression profile, which includes the expression of various genes such as the pleiotropic cytokines IL-6, IL-1α, IL-1β, and IL-8. In addition, the transcription factor C/EBPbeta collaborates with IL-6 to enhance initiation of the pro-inflammatory cascade, as demonstrated in cells carrying B-RAFV600E and H-RAS G12V mutations.^287^

#### Chemotherapy-induced senescence (CIS)

Chemotherapy-induced cellular senescence is a commonly used cellular model. Drugs like doxorubicin can induce cells to enter a senescent state.^288^ In this model, cells treated with doxorubicin display characteristic features of senescence. For instance, the expression of 4-HNE and GPX4 increases, while SIRT1 expression decreases. Furthermore, these senescent cells exhibit elevated levels of pro-inflammatory cytokines like IL-6, IL-17, and TNF-α, along with reduced levels of the anti-inflammatory cytokine IL-4, indicating the presence of inflammation.^289^ Similarly, treatment of melanoma cells with Palbociclib leads to cell cycle arrest at the G0/G1 phase, accompanied by SA-βgal and SASP that includes factors such as IL-6, IL-8, and CXCL1.^290^ In addition, doxorubicin-induced senescence in H9c2 myocardial cells results in increased expression of 4-HNE and GPX4, decreased SIRT1 expression, and heightened levels of pro-inflammatory cytokines (IL-6, IL-17, and TNF-α), while the anti-inflammatory cytokine IL-4 is reduced.^291–293^ Furthermore, primary human astrocytes exposed to X-rays exhibit increased expression of senescence-associated proteins (p16INK4a and Hp1γ) and cytokines associated with SASP, such as IL-1β and IL-6.^294–296^ Further details of other in vitro models are shown in Table 2.

**Table 2:** Multiple models to study aging

Model type	Model name	Description
*In vitro models*		
Replicative senescence (RS)	Replicative senescence model^375,376^	Reduced saturation density, heightened cell surface area and volume, cell cycle arrest, shortened telomeres, and an increased occurrence of SA-β-gal positive staining
Chemotherapy-induced senescence (CIS)	hydroxyurea induced model^377^	Increased ROS and SA-β-gal positive staining and decreased cell proliferation
	Aβ1-42 oligomers model (AD model)^378,379^	Increased ROS and SA-β-gal positive staining, PAI-1 and p21 mRNA levels, and decreased SIRT1
	D-galactose model^299^	Increased ROS, SA-β-gal positive staining, inflammation level, P16, P21 and P53 genes, and decreased of NRF2 and HO-1
	Doxorubicin (a DNA topoisomerases inhibitors)^380,381^	Cell cycle arrest, DNA damage, telomere shortening, increased expression of p16lnk4a
	Palbociclib treated (a CDK4/CDK6 inhibitor)^382^	G0/G1 arrest, growth arrest, reduced Rb expression, increased SA-β-gal positive staining, and IL-6, IL-8, CXCL1 secretion
Stress induced premature senescence (SIPS)	X-ray induced model^383,384^	Irreversible G1 cell cycle arrest, DNA damage, increased SA-β-gal positive staining, IL-1β, IL-6, IL-8 and other SASP cytokines
	UVB induced model^385^	Growth arrest, increased SA-β-gal positive staining, senescence-associated gene overexpression, deletion in mitochondrial DNA
	H_2_O_2_ induced premature senescence^386,387^	Increased SA-β-gal positive staining, irreversible G1 cell cycle arrest, telomere shortening, and increased p21 and gadd45 expression
	Tert-butylhydroperoxide (t-BHP) induced premature senescence model^388^	Growth arrest, increased SA-β-gal positive staining, the presence of the common 4977-bp mitochondrial deletion, overexpression of p21waf-1 and the subsequent inability to phosphorylate pRb, increased senescence-associated genes expression
	Ethanol induced premature senescence model^389,390^	Growth arrest, increased SA-β-gal positive staining, overexpression of p21waf-1 and the subsequent inability to phosphorylate pRb, the presence of the common 4977-bp mitochondrial deletion, increased senescence-associated genes expression
	Hyperoxia induced model^391,392^	Irreversible G1 cell cycle arrest, telomere shortening, increased protein degradation, increased lipofuscin/ceroid formation, and accumulation
Oncogene-induced senescence (OIS)	Mos-overexpression model^393^	The growth arrest DNA damage, upregulation of p16INK4a, and increased SA-β-gal positive staining
	B-RAF V600E model^394,395^	Cell cycle arrest, upregulation of p16INK4a, and increased SA-β-gal positive staining
	H-RAS G12V model^396,397^	Upregulation of p16INK4a, low phosphorylated Rb, increased SA-β-gal positive staining
DNA methyltransferases inhibitor	5‐aza‐2′‐deoxycytidine induced model	Growth inhibition, increased SA‐β‐gal,^398^ increased p16, decreased p53^399^
Telomerase activity inhibitor	SYUIQ-5 induced model	Growth inhibition, increased SA‐β‐Gal, increased p16, p21, p27^400^
	Cyclin E overexpression model^401,402^	Cell cycle arrest, DNA damge, and increased SA-β-gal positive staining
	IMR90 ER: RAS model^403^	The growth arrest, increased SA-β-gal positive staining and SASP markers
Elder donors derived aging models	iPSC-derived neuron with senescence phenotype^404,405^	iPSC-derived neurons from elder donors have senescence-related gene expression
	Induced neuron with senescence phenotype^406,407^	Induced neurons from fibroblast of elder donors have age-dependent transcriptomic signatures
*In vivo models*		
SAMP models	SAMP6 (senile osteoporosis model)^408–410^	Decreased bone formation, and increased bone marrow adiposity, proliferator activator γ (PPARγ), and crimp-related protein 4 (Sfrp4)
	SAMP8 (AD model)^301^	Age-related learning and memory deficits, amyloid-β deposition, abnormal autophagy activity
	SAM10 (neurodegenerative disease model)^411^	Spontaneous brain degeneration leading to impairments in learning and memory as well as emotional disturbances
Physicochemical induced aging model	D-Galactose induced model^299,412^	Increased ROS, SA-β-gal positive staining, inflammation level, apoptosis, up-regulations of P53 and P21 genes expressions, and mitochondrial dysfunctions
	D-galactose and AlCl3 induced AD model^413^	Memory deficit, neuronal damage and caspase-3 overexpression in the hippocampus
	D-galactose and NaNO2 induced AD model^414^	Increased oxidative stress, neuronal damage in the CA1, CA3, and CA4 regions of the hippocampus, impaired cognitive function, memory impaired, deterioration of sperm quality and testicular morphology
	AlCl3 induced model^413,415^	A notable decline in cognitive function characterized by impaired short-term memory, heightened anxiety, and a decline in spatial and reference memory
	iron radiation induced model^416^	Increased SASP marker, SA-β-Gal, IL-8 and persistent DNA damage responses
	O3 induced model	Including thymic atrophy, decreased body weight and exploratory activity, and increased oxidative damage
Premature aging models (WS)	Wrn−/− Terc−/− model^417^	Changes associated with aging include the shortening of telomeres, the onset of hair graying, alopecia, cataracts, malignancies, osteoporosis, and type II diabetes
	WrnΔhel/Δhel model^412^	Severe cardiac interstitial fibrosis, insulin resistance, hypertriglyceridemia, increased ROS, oxidative DNA damage, cancer incidence, and shortened lifespan
Premature aging models (HGPS)	LmnaL530P/L530P model^418^	Severe growth retardation, hair loss, osteoporosis, muscle atrophy
	LmnaHG/+ model^419^	Slow growth, osteoporosis, hair loss, partial fat malnutrition
	LmnaG609G/G609G model^420^	Infertility, weight loss, growth retardation, spinal curvature, calcification of blood vessels, decreased bone density, and insulin-like growth factor
	Zmpste24-/- model^421^	Dilated cardiomyopathy, lipodystrophy, muscular dystrophy, severe growth retardation, and premature death,
Other premature aging phenotype	BubR1H/H model^422^	Gliosis in the brain, arrhythmias, cataracts, hunchbacks, lipodystrophy, thinning of the skin, impaired vascular elasticity and fibrosis, and shortened life expectancy
	Ndufs4−/− model (a progressive neurodegenerative phenotype with leigh syndrome)^423^	Lethargy, ataxia, weight loss, premature death
	ERCC1−/− or Δ/− model^424,425^	Growth retardation, ataxia, loss of visual acuity, cerebellar hypoplasia, encephalopathy, kidney failure, proteinuria
	Sod1−/− model^426^	Muscle atrophy, fat metabolism disorders, hearing loss, cataracts, thinning of the skin, and defects in wound healing
	Klotho−/− model^427,428^	Arteriosclerosis, cardiovascular injury, infertility, short lifespan, skin atrophy, osteoporosis, and emphysema
	XpdTTD/TTD model^429^	Early graying, osteoporosis, cachexia, kyphosis, osteosclerosis, sterility, and shortened lifespan
	PolG model (mutation in mtDNA Polγ)^430,431^	Alopecia, anemia, weight loss, hearing loss, reduced bone mineral density, and cardiomyopathy
	PolgAmut/mut model^432^	Anemia, enlarged heart, osteoporosis, spine curvature, and reduced fertility
	Nfkb1−/− model^303^	Shortened lifespan, kyphosis, osteoporosis, tissue inflammation, and gliosis of the central nervous system
	Terc−/− model^433,434^	Shortened lifespan, reduced fertility, tissue atrophy, and impaired organ functions
	3xTg-AD model (AD model)^435,436^	Memory impairment, cognitive deficits, synaptic dysfunction, abnormal hyperexcitation of hippocampal neurons, amyloid plaques, and p-Tau accumulation
	Tg2576 model (AD model)^437,438^	Cognitive impairment, memory loss, oxidative lipid damage and inflammation in the brain
Longevity models	Naked mole-rats^439,440^	As they age, no significant increase in mortality is observed and they retain basic physiological function. They age with health and this anti-aging properties make them as a good model for aging research.
	Planarians^309^	With significant regenerative powers, planarians are considered immortal, DNA efficient repair mechanism, strong telomerase activity
	Salamander	Strong ability to regenerate, clearance of senescent cells,
	Turtle	Clearance of ROS, strong telomerase activity, Efficient DNA repair mechanism
Transgenic delayed aging model	Ames dwarf mice (Prop1df/df)	Prop1 gene recessive point mutation, impaired pituitary development smaller body size, PI3K/Akt/mTOR pathway downregulation^441^
	Snell dwarf mice (Pit1dw/dw)	Pit1 gene spontaneous mutations, impaired pituitary development, smaller body size, reduced immunosenescence^442^

*AD* Alzheimer’s disease, *WS* Werner syndrome, *HGPS* Hutchinson-Gilford progeria syndrome, *ERCC1*−/− or *Δ/−* represents mutation and/or deletion in the Ercc1 gene, *UVB* ultraviolet B, *B-RAF V600E* valine is substituted for glutamic acid, *IMR90 ER* RAS model: IMR90 human primary fibroblast is infected with a 4-hydroxy-tamoxifen (4-OHT) inducible ER:RAS construct, where H-RASG12V is fused to a mutant form of the estrogen receptor (ER) ligand binding domain. *PolgAmut/mut* mutations in mtDNA PolgA, *LmnaHG/+* farnesylated uncleaved form of LMNA, *SAM* senescence-accelerated mouse, *SAMP* senescence-prone inbred strains, *SAMR* senescence-resistant inbred strains, *SASP* senescence-associated secretory phenotype, *WrnΔhel/Δhel* helicase mutation in Wrn gene, *XpdTTD/TTD*: mutation of Xpd gene in the human disorder trichothiodystrophy (TTD), *BubR1H/H* mutations in BubR1 hypomorphic alleles

### In vivo models

The mouse has quickly emerged as the preferred mammalian model organism in aging research. This is primarily attributed to several factors, including its relatively short lifespan compared to humans, the close similarity of its genome and physiology to humans, and the ease with which its genetics can be manipulated, including the availability of various mutant strains. These advantages make mice an excellent model for studying the aging process and investigating potential interventions and treatments for age-related conditions.^297^ Mouse models of accelerated aging involve physically induced models (e.g., radiation and O3), chemically induced models (e.g., D-galactose and D-galactose-combined therapy), “senescence-prone” mice (e.g., SAMP), and premature aging models (e.g., HGPS) (Table 2).

#### Induced or genetic aging models

In physically induced models, the inhalation of ozone is a frequently employed technique for inducing premature senescence. When male BALB/c mice are exposed to ozone at a concentration of 1.2 mg/m^3^ for 10 h per day, they exhibit thymic atrophy and an elevated level of oxidative damage. Subsequently, there is a decline in the immune function of the mice, which is closely associated with oxidative stress. This decline is characterized by an increase in IL-6 levels, reduced splenocyte proliferation, decreased production of IL-2, diminished natural killer (NK) cell activity, and a weakened antigen-specific response.^298^

For chemical induction models, the D-galactose-induced aging model is widely preferred in chemical induction studies due to its convenience, higher survival rate, and minimal side effects. This model effectively mimics aging in vivo by inducing changes in various tissues and organs. When mice are treated with certain concentrations of D-galactose, they exhibit increased levels of ROS and inflammatory cytokines such as NOS-2, IL-1β, IL-6, TNF-α, and NF-κB. These alterations contribute to the aging-like phenotype observed in this model. Moreover, researchers have developed combined methods involving D-galactose to induce premature aging. For instance, the D-galactose and AlCl3 model and the D-galactose and NaNO2 model are commonly used. These combinations enhance the aging effects and provide additional insights into the mechanisms underlying accelerated aging.^299^

Senescence-accelerated mouse/prone (SAMP) strains, specifically the SAMP1/Yit substrain, have been recognized as valuable models for studying the genetic aspects of aging. In particular, the SAMP1/Yit mice have been used as a model for Crohn’s disease, which is a chronic and recurring inflammatory bowel disease. The mice exhibit both acute and chronic inflammation in the ileum and cecum, displaying a non-continuous pattern of inflammation.^300^ On the other hand, the SAMP8 mouse strain is considered an excellent model for investigating Alzheimer’s disease (AD), a cognitive decline disorder that predominantly affects the elderly. The SAMP8 mice exhibit reduced expression and lower activity of anti-aging factors including silent information regulator type (sirtuin/Sirt), Forkhead box class O (FoxOs), and Klothos. These factors play crucial roles in the aging process.^301^

Aging models with apparent inflammation phenotypes include Nfkb1 deficient mice (Nfkb1−/−). Nfkb1−/− mice have shortened lifespan, kyphosis, osteoporosis, tissue inflammation, and gliosis of the central nervous system.^302^ Interestingly, the accumulation of senescent cells with telomere-dysfunction in Nfkb1−/− tissues can be effectively hindered through the implementation of anti-inflammatory or antioxidant treatment in mice.^303^ This noteworthy observation underscores the promising utilization of these mice as valuable models for investigating age-related interventions. Details of other in vivo aging models are also shown in Table 2.

#### Premature aging models

Premature aging models can be categorized into two main types: progeroid syndrome models and other models exhibiting premature aging phenotypes. Progeroid syndromes are exceptionally uncommon human disorders characterized by early onset aging and a reduced lifespan. These syndromes include laminopathies, such as Hutchinson-Gilford progeria syndrome (HGPS), which disrupt the balance of the nuclear envelope, as well as conditions that affect telomere length and DNA repair mechanisms, like Werner syndrome and Cockayne syndrome.

The identification of specific mutations causing these syndromes has enabled the development of mouse models that simulate premature aging.^304^ For instance, mouse models of HGPS have been created by modifying the Lmna gene or its processing enzyme. Among these models, the LmnaG609G mouse model (featuring the mutation 1827C > T; Gly609Gly) closely resembles human phenotypes.^305^ This includes heightened inflammation markers like IL-6, caspase 1, and Nlrp3, increased oxidative stress, persistent DNA damage, and cell cycle arrest.^306,307^ In addition, two independently generated mouse models deficient in the Zmpste24 gene (Zmpste24 − /− mice) also exhibit elevated expression of caspase 1 and Nlrp3, severe growth retardation, dilated cardiomyopathy, muscular dystrophy, lipodystrophy and premature death.^307,308^

#### Classic longevity animal model

Many animals naturally have long lifespans. Decoding the underlying mechanisms for provide insights for developing anti-aging strategies. One such remarkable example is the naked mole rat, a socially oriented mammal that dwells in subterranean burrows. This extraordinary creature holds the esteemed distinction of being the longest-living rodent, with a maximum lifespan that surpasses an astonishing 30 years.^309^ With aging, naked mole rats will not lose their physiological functions, and their mortality will not increase significantly.^310^ As a successful aging specifications, nude mole also achieved resistance to tumor through a variety of methods, such as efficient DNA damage repair, synthesis of unique anti-inflammatory high molecular weight hyaluronan.^311^ The special longevity mechanism of naked mole makes it a good animal model for longevity research and provides a blueprint for exploring the strategies of delaying human aging.

Planaria is a type of flatworms. For planarians, small fragments of almost any tissue can be regenerated into an entire individual. This has led it to be considered immortal. The shortening of telomeres during cell division is a major obstacle to infinite cell division, and planarians overcome this problem by upregulating telomerase expression during regeneration.^312^ On the other hand, planarians also achieve resistance to tumors through a variety of means, such as efficient DNA repair mechanisms.^313^ All of these mechanisms provide enlightenment for us to study human aging.

As an amphibian, the salamander is also very long-lived. Their limbs and many of their organs are capable of regenerating. Axolotls age very slowly, and their phenotypes are less pronounced. Its physiological mechanism to clear senescent cells in time prevents senescent cells from accumulating in its body, which may explain its slow aging.^314^ The salamander’s large genome may have provided lines of defense for potentially harmful mutations, such as regulators. This also laid the foundation for the prevention of tumors.^315^

Turtles are a typical group of long-lived animals. The protection of the carapace keeps them from predators. During diving, turtles are chronically deprived of oxygen, which allows them to upregulate glutathione-related enzymes to clear away ROS.^316^ Inhibition of ROS and unique telomerase and perhaps DNA repair mechanisms make turtles’ longevity possible.^317^

### Centenarian human models

Unlike artificial models, the centenarian model, as a naturally occurring model, has become an indispensable tool for human beings to decipher longevity. Unlike other people, centenarians have significantly different levels of hormones, cholesterol, etc. in their bodies. Inflammation levels in centenarians show a better balance compared to others, and therefore inflammation levels have been used to predict healthy lifespan. The offspring of centenarians also typically maintain lower levels of chronic inflammation in their bodies, and these lower levels can increase as the centenarian ages, but ultimately those who can maintain lower levels of inflammation may have the best chance of maintaining their bodies in good health. This suggests that genes may be a key factor in maintaining low levels of inflammation in centenarians.^6^ In addition, the low susceptibility of centenarians to diseases raises the question of whether their immune system is stronger. Studies have shown lower levels of B cells, similar numbers of T cells, and a higher percentage of cytotoxic T cells in the peripheral blood of supercentenarians.^318^ Compared with animal models, the centenarian model can more realistically reflect the changes in human organs as well as peripheral blood components and has indispensable reference value for human anti-aging research. In conclusion, the long-lived elders are the result of “natural experiments”. They show us that it is possible for individuals to live longer and healthier lives, even if they are influenced by risky genes or if they choose to ignore health information on their own.

## Cutting-edge single-cell technology

Single-cell technology reveals organismal activity at the level of the genome, epigenome, transcriptome, proteome, and metabolome. A recent achievement of single-cell technology in the aging field is an aging atlas in different species at a multi-omics level, which allows us to understand aging with a more systemic view.

Aging is extremely heterogeneous, especially from a transcriptome perspective.^198^ The aging transcriptome landscape in mouse, rat, and cynomolgus monkey is presented in Table 3, including samples, time, platform, cell numbers, and main conclusions. In the future, spatial single-cell technologies (spatial transcriptome and spatial metabolome) make it possible to construct three-dimensional aging atlases at the organ level.^319^

**Table 3:** Single cell landscape of aging in mouse, rat and cynomolgus monkey

	Sample &Time & Platform and cell number	Conclusions
*Mouse*		
2018, Tabula Muris Consortium^443^	(1) Brain, Thymus, Bone marrow, Kidney, Liver, Spleen, Lung, Small intestine, Peripheral blood; (2) 19 male and 11 female mice at 1, 3, 18, 21, 24, 30 months; (3) 10x Genomics (356,213 cells)	Molecular changes associated with the key hallmarks of aging can be observed in various tissues and cell types. For instance, in leukocytes, which are white blood cells, specific molecular alterations reflect the aging process. Old leukocytes often exhibit increased expression of pro-inflammatory markers and decreased expression of anti-inflammatory markers, indicating a shift towards a pro-inflammatory state.
2020, Tony Wyss-Coray group^444^	(1) Brain, Bone marrow, Kidney, Liver, Spleen, Lung, Intestine, Skin, Brown adipose tissue, WBC etc. (17 organs); (2) 4 male (1–27 months old) and 2 female (1–21 months old) mice (interval of 3 months); (3) Bulk RNA and plasma proteomics at 10 ages	Asynchronous inter- and intra-organ progression model of aging suggests that the aging process is not uniform across organs and tissues and can occur at different rates. Furthermore, widespread activation of immune cells becomes particularly prominent during aging. This immune cell activation is initially noticeable in white adipose depots during middle age, involving various immune cell types, including T cells, B cells, and plasma cells that express immunoglobulin J chain.
*Rat*		
2020, Guang-Hui Liu Group^445^	(1) White adipose tissue, Brown adipose tissue, kidney, skin, liver, aortic tissues were isolated from randomly selected Y-AL; (2) Young ad libitum: 3 male and 3 female rats (5 months old); Old ad libitum: 2 male and 4 female rats (27 months old); Old caloric restriction: 3 male and 3 female rats (27 months old) animals; (3) 10x Genomics (166,111 cells)	The inflammatory response tends to increase with age. However, it has been observed that caloric restriction can have a systemic repressive effect on this inflammatory response. Then, abnormal cell-cell communication patterns are commonly observed during aging, particularly an excessive interplay of pro-inflammatory ligands and receptors. Lastly, interventions targeting metabolism have the potential to impact the overall immune response. These metabolic interventions may have a global effect on modulating the immune system during the aging process.
*Cynomolgus monkey*		
2020, Guang-Hui Liu Group^446^	(1) Ovaries; (2) four juvenile (4, 5, 5, and 5 years old) and four aged (18, 19, 19, and 20 years old) cynomolgus monkeys; (3) STRT-sequencing (2601 cells)	The disruption of antioxidant signaling in early-stage oocytes and granulosa cells is a clear indication of oxidative damage as a critical factor in the decline of ovarian function with age. This oxidative damage is a significant contributor to the deterioration of the ovaries as it affects the delicate balance between ROS production and the antioxidant defense system. The impairment of antioxidant signaling pathways in these specific cell types further accentuates the susceptibility of the ovary to oxidative stress, leading to a decline in reproductive capacity and fertility as women age.
2021, Guang-Hui Liu Group^447^	Lung (*n* = 15) and heart (*n* = 6) monkeys (young 4–6 years old) and 8 old 18–21 years old). 10× Genomics single nucleus RNA-seq (109,609 for lung, 42,053 for heart)	Increased systemic inflammation is a hallmark of cardiopulmonary aging. With age, the expression of the SARS-CoV-2 receptor angiotensin-converting enzyme 2 (ACE2) is elevated in the pulmonary alveolar epithelial barrier, cardiomyocytes, and vascular endothelial cells. In addition, in aged cardiopulmonary tissues, there is an accumulation of IL-7, which induces ACE2 expression. Besides, it has been found that vitamin C can block the IL-7-induced ACE2 expression.
2020, Jing Qu group^448^	Aortic and coronary arteries from 8 young (4–6 years old) and 8 old (18–21 years) monkeys modified STRT-seq (8122 cells)	A comprehensive understanding of primate aortic and coronary vasculature aging at the single-cell level reveals the key role of FOXO3A downregulation in driving vascular dysfunction. This research provides insights into the molecular mechanisms underlying age-related changes in the primate vascular system. The study highlights that the downregulation of FOXO3A, a transcription factor involved in cellular stress response and longevity, plays a significant role in the development of vascular dysfunction associated with aging. The findings shed light on the specific molecular alterations occurring in the vasculature and emphasize the importance of FOXO3A as a potential therapeutic target for age-related vascular diseases.
2021, Jing Qu Group^449^	Neural retina and RPE-choroid layers from 8 young (4–6 years old) and 8 old (18–21 years) monkeys STRT-seq (total 7461 cells)	Oxidative stress is a prominent hallmark of aging in the neural retinal layer, while an intensified inflammatory response characterizes RPE and choroidal cells. Among the retinal cells, RPE cells are particularly susceptible to aging, exhibiting greater vulnerability compared to other cell types in the retina.

## Intervention strategies in aging

### Lifestyle interventions

A healthy lifestyle has long been recognized as the most effective way to maintain health and fight aging.^320^ More and more research has proven that maintaining a healthy lifestyle, such as adequate nutrition,^321^ moderate exercise,^322^ and good mental state^323^ can effectively delay aging. Balanced and adequate nutrition intake has a positive effect on aging. Many of the nutrients that people take in, such as minerals, probiotics, etc., play an important role in alleviating inflammation and regulating immunity. Long-term polyphenyl-rich dietetic pattern has been proved to improve intestinal permeability and the level of inflammatory markers.^324^ Previous studies have also proved that the intake of probiotics, such as Lactobacillus pentosus var. plantarum C29, has been proved to significantly reduce the level of systemic inflammatory factors and the expression of aging markers p16 and p53.^325^ Similarly, consumption of polyunsaturated fatty acids has been shown to significantly reduce levels of inflammatory cytokines throughout the body^326^ The continuous intake of vitamins, such as vitamin C and vitamin E, can effectively improve the function of immune cells in the elderly, Such as the chemotaxis and phagocytosis of neutrophils.^327^ The supplementation of minerals, such as zinc, can increase the naive T cell subgroup^328^ and improve the homeostasis of Th1 and Th2 cells.^329^ These all emphasize the importance of maintaining balanced nutrition intake.

Exercise is an efficient strategy for delaying aging^330^ through various mechanisms, such as DNA damage^331^ and oxidative stress.^332^ Recent study found that middle-aged marathon/triathletes had higher telomerase activity and longer telomere length in circulating white blood cells compared to the control group.^333^ Correspondingly, resistance training for five months in older overweight or obese women reduced the number of P16-expressing cells in their thigh fat tissue.^334^ These evidence suggest that exercise can effectively reduce the appearance of age-related markers, such as p16, so as to achieve the effect of delaying aging.

Keeping a good mental state can also delay aging. Psychological stress affects neuroendocrine function through hypothalamus-pituitary-adrenal axis.^335^ The continuous activation of this circuit leads to the continuous increase of glucocorticoid level, which will lead to hippocampal atrophy, a phenomenon closely related to aging.^323^ In addition. A longitudinal study found that the elevation of inflammatory markers increased in elderly people with high self-reported stress levels during follow-up. This may reflect the internal relationship between psychological stress and inflammation.^336^ These findings suggest that improving mental health and alleviating psychological stress have a positive effect on aging. Lifestyle choices are closely associated with ageing. Keeping a healthy life sometimes means a longer life.

### Anti-inflammation strategies

Recent studies have demonstrated that the pro-inflammatory cytokine network is one potential anti-aging target, using anti-inflammation drugs such as metformin, aspirin, rapamycin, and ibuprofen. For example, metformin can reduce chronic inflammation and improve healthy mid-life aging by acting on possible targets such as IKK/NF-κB in patients with Type 2 diabetes,^337^ as well as GPX7/NRF2^338^ and a recently found target, PEN2.^339^ Aspirin can postpone the occurrence of replicative senescence by decreasing oxidative stress. The latest studies have identified CD36 as key to SASP-related mechanisms and reduced SASP secretion in senescent cells by silencing CD36 in senescent muscle tissue cells using CD36-specific short interfering RNA.^340^ In general, current strategies of anti-inflammation and immune enhancement, including potential targets and main adaptation diseases are summarized in Table 4.

**Table 4:** Current intervention strategies against aging

Strategies	Possible targets	Development status	Details
Dasatinib (D) and Quercetin (Q)^450,451^	Pan-receptor tyrosine kinases; PI3K isoform and Bcl-2 family members	Phase II clinical trial (NCT02848131) for chronic kidney disease^346^	Dasatinib has been shown to effectively eliminate senescent human fat cell progenitors. On the other hand, quercetin has demonstrated greater efficacy against senescent human endothelial cells and mouse BM-MSCs. When used in combination (D + Q), these two drugs induce apoptosis more efficiently by targeting a larger number of senescent cell-associated phenotypes (SCAPs) compared to either drug used alone. This synergistic effect has been observed in various types of senescent cells.
Metformin^365,452,453^	IKK and/or NF-κB; AMP-activated protein kinase activity; Glutathione peroxidase 7 (GPx7) and nuclear factor erythroid 2-related factor 2 (Nrf2)	Approved for type 2 diabetes^454,455^	Metformin exerts its effects by preventing the translocation of NF-κB to the nucleus and inhibiting the phosphorylation of IκB and IKKα/β. In addition, it enhances the activity of AMP-activated protein kinase, leading to reductions in both the accumulation of oxidative damage and chronic inflammation. Metformin upregulates the endoplasmic reticulum localized GPX7 and increases the nuclear accumulation of Nrf2. Metformin-Nrf2-GXx7 pathway delays aging.
Rapamycin^456^	mTOR	Approved by the FDA for the prevention of organ rejection following kidney transplantation and the treatment of lymphangioleiomyomatosis^457–459^	The TOR (target of rapamycin) kinase restricts longevity through mechanisms that are not fully understood. Rapamycin, a compound that inhibits the mammalian TORC1 complex responsible for regulating translation, has been shown to increase lifespan in various species, including mice. Our research demonstrates that rapamycin specifically attenuates the inflammatory characteristics exhibited by senescent cells.
Fisetin^460,461^	Glutathione; mitochondria; Key neurotrophic factor signaling pathways; Lipoxygenases and pro-inflammatory eicosanoids and their by-products	Preclinical animal models	Fisetin exhibits direct antioxidant activity and enhances intracellular levels of glutathione, the primary antioxidant within cells. Furthermore, it preserves mitochondrial function when faced with oxidative stress. In addition, it inhibits lipoxygenase activity, thereby decreasing the production of pro-inflammatory eicosanoids and their derivatives. Overall, fisetin helps mitigate the effects of age-related neurological disorders on brain function.
Curcumin^462,463^	Nrf2 and NF-κB pathways	Preclinical animal models	Curcumin has been found to exhibit senolytic properties against senescent human intervertebral disc (IVD) cells by downregulating the Nrf2 and NF-κB pathways. The application of curcumin and o-Vanillin effectively eliminated senescent IVD cells and mitigated the SASP, which is linked to inflammation and back pain. This research highlights the potential of curcumin in targeting senescent cells and alleviating the negative consequences associated with senescence in the intervertebral discs.
Piperlongumine^464–467^	Oxidation resistance 1 (OXR1); NF-κB signaling pathway	Preclinical animal models	Piperlongumine exerts its effects through two distinct mechanisms. Firstly, it selectively induces the death of senescent cells by directly binding to oxidation resistance 1 (OXR1), resulting in its degradation through the proteasomal pathway. This degradation of OXR1 leads to an increase in ROS production within the cells. Secondly, Piperlongumine inhibits the growth of lung tumors by targeting the NF-κB signaling pathway. Overall, these actions of Piperlongumine demonstrate its potential as a promising therapeutic agent for targeting senescent cells and inhibiting tumor growth in the lungs.
Aspirin^468–470^	NO; Telomerase	Approved	Aspirin has been shown to inhibit senescence and SASP, providing a potential mechanism for its beneficial effects on healthy aging. For instance, aspirin has been found to delay the onset of replicative senescence in endothelial cells. This effect is achieved by increasing nitric oxide synthesis and reducing oxidative stress, ultimately leading to the upregulation of telomerase activity.
Fenofibrate^347^	PPARα	Preclinical animal models	In investigations involving human osteoarthritis and aging primary chondrocytes, the administration of fenofibrate exhibited several beneficial effects. Specifically, fenofibrate treatment upregulated the expression of PPARα, resulting in reduced chondrocyte senescence. Moreover, it increased autophagic flux and induced the selective elimination of senescent cells.
CAR-T/NK	uPAR; FAP	Preclinical animal models	CAR-T cells targeting uPAR and FAP can effectively clear senescent cells, while CAR-T cells targeting FAP can further reduce the extent of cardiac fibrosis. Adoptive NK cell infusion also successfully reduced senescence markers and SASP levels.

*IKK* IkappaB kinases, *NF-κB* nuclear factor kappa-B, *AMP* adenosine monophosphate, *GPX7* glutathione peroxidase 7, *NRF2* nuclear factor erythroid 2-related factor 2, *PEN2* PENETRATION2, *Nrf2* nuclear factor E2-related factor 2, *SASP* senescence-associated secretory phenotype, *OXR1* oxidation resistance 1, *ROS* reactive oxygen species, *NO* nitric oxide, *NMDA* N-methyl-D-aspartate, *MAPK* mitogen-activated protein kinase, *ERK1/2* extracellular signal-regulated kinase 1/2, *IFN* interferon, *CDK4/6* cyclin-dependent kinases 4 and 6, *PI3K* phosphatidylinositol-4,5-bisphosphate 3-kinase, *SCAPs* senescent cell anti-apoptotic pathways, *PPARα* peroxisome proliferator-activated receptor alpha, *MSC* mesenchymal stem cell

### Senolytic drug for eliminating senescent cells

Of all the anti-ageing cell therapies, Senolytics (removal of senescent cells) are the most well-developed and specific but also the most controversial. Since 2015, several Senolytics have gone from identification to clinical trials. The first Senolytics-like drug combination was Dasatinib and Quercetin. Dasatinib removes senescent human adipocyte progenitor cells, while Quercetin is more potent in killing senescent human endothelial cells and bone marrow stem cells in mice.^341^ The most potent removal of senescent cells was achieved when these two compounds were combined. In several mouse experiments, the treatment alleviated inflammation^342^ and age-related diseases of the intestines^343^ and bone.^344^ In the first human trial, ‘Dasatinib + Quercetin’ treatment increased patients’ 6-minute walking distance by an average of 21.5 meters. However, other indicators including Pulmonary function, clinical chemistries, frailty index (FI-LAB), and reported health did not change significantly.^345^ Further research is needed on the core indicators such as SASP levels which will also predict efficacy and side effects over time. In clinical trials in patients with diabetic nephropathy, Dasatinib, and Quercetin significantly reduced senescent cell levels as well as circulating SASP levels over 11 days.^346^ Considering the potential toxicity of the inflammation produced by cell death and grab, there is a lack of long-term follow-up studies for this therapy and the current evidence is insufficient to support its long-term anti-aging effectiveness. In addition, another Senolytic called Fenofibrate can induce selective elimination of senescence cells through upregulating PPARα expression.^347^ Excessive clearance of senescent cells (especially for senescent liver sinusoidal endothelial cells or adipocytes) has also been reported to accompany the dilemma faced by cells that cannot be replenished in time, although Quercetin + Dasatinib clears mainly p16 macrophages.^348^

### (CAR-)T/NK for eliminating senescent cells

On the other hand, immune cell-mediated clearance of senescent cells is emerging as a promising strategy to fight multiple chronic diseases and aging.^349^ It was found that anti-uPAR-CAR-T cells were effective in removing senescent cells in vitro and pre-cancerous and malignant cells in mouse models of liver and lung in the presence of potentially toxic.^350^ Previously, CAR-T therapy targeting FAP was found to significantly reduce cardiac fibrosis and improve heart function. Surprisingly, FAP-CAR-T cells did not target other normal cells in the body and did not cause recruitment and infiltration of immune cells and increased levels of inflammatory factors, such as IL-1 and IL-6.^351^ In addition, clearance of senescent cells by NK cell-based immune cells prolongs the lifespan of mice and appeared to be safer.^352^ In the aged mice and healthy/obese volunteers, adoptive NK cell infusion significantly reduced senescence markers and SASP levels without significant toxic side effects.^353,354^ In addition, their combination with the immunomodulatory factor Acein significantly decreased the expression of tissue senescence markers and age-related genes in a mouse model of senescence.^353^

### Stem cell therapy

Stem cell therapy has become an effective strategy for the treatment of aging-related diseases. Many studies have shown that the number and function of different somatic cell populations declines with age, which diminishes the regenerative potential of tissues and organs. However, there is limited evidence that loss of stem cell function is a major driver of age-related pathology and shortened lifespan.^355–358^ Trials have found that after stem cell injections in aging debilitated patients, many symptoms improve, and inflammatory marker levels decrease.^359^ MSC transplantation for acute stroke improves patient symptoms and promotes neurological recovery in ischemic stroke patients without adverse effects.^360–362^ Meanwhile, mesenchymal stem cell transplantation for Parkinson’s disease can significantly improve the daily activities and motor functions of Parkinson’s disease patients without side effects, as well as being safe and reliable.^363,364^ In addition, HSC transplantation is a very rapidly and effective clinical treatment for age-related diseases such as AML.

### Organ regeneration and transplantation

Similar to stem cell transplantation, organ transplantation is a very effective anti-aging modality because it can “repair” the damage caused by aging in the most simple and brutal way. For example, many risk indices for age-related diseases were improved after thymic regeneration in humans (taking three commonly used drugs: growth hormone, dehydroepiandrosterone, and metformin),^365^ and biological age was reversed.^366^ However, in order to use this technology in the field of anti-aging, two problems must first be solved: the shortage of spare organ stock and rejection after transplantation. Currently, most donated organs come from relatives, cerebrally dead donors, or even animals. For organs from old donors, Senolytics are used to rejuvenate aging organs.^367^ Meanwhile, the world’s first “transplantation” of a porcine heart into a human was initially successful and has not caused hyperacute rejection in humans. If animal organs are successfully transplanted and the cost is controlled, human disease treatment and anti-aging will take a step forward.

Resistance and reversal of aging is the ultimate goal of aging research. Anti-inflammation, removal/improvement of senescent cells, stem cell therapy and organ transplantation have become crucial ways to reverse aging in humans.

## Discussion

The global population has been experiencing an aging trend, and the elderly population is more susceptible to infections, increased mortality, and morbidity.^368,369^ Chronic inflammation appears to be closely linked to aging, and this review focuses on inflammaging, providing an overview of the molecular, cellular, and organ levels of the human body. At the cellular level, external DAMPs activate different immune cells, promoting inflammation and leading to immunosenescence. Dysfunctional immune cells cannot clear senescent cells in a timely manner, leading to inflammation and the development of aging-related diseases and/or normal aging in different organs (Fig. 1). In addition to the separate analysis of molecules, cells, and organs, we also hope to closely link the cells, organs and molecule with single-cell multi-omics (including genome, transcriptome, epigenome, proteome and metabolome) and spatial omics.

The construction of animal models is an effective way to study aging (Table 2). The present review systematically summarizes the characteristics of different aging models, both in mice and in vitro cell lines. To date, an aging atlas of the whole organ has been profiled in mice. Considering the differences between different species,^370^ it will be important to construct non-human primate and human aging models in the future. In addition, several resource tools have been developed to assist in aging research, such as SeneQuest to promote the discovery of genes associated with senescence (available at http://Senequest.net).^371^ On the other hand, other technologies, such as machine learning which integrates multi-modal and multidimensional big data, are also important to address the complexity of aging problems (Table 4). A recent paper summarizes the development and application of aging clocks with the help of machine learning analysis of histological data. This illustrates the ability of machine learning to identify novel biomarkers of biological aging and provides a boost to early warning and intervention in aging and precision medicine strategies.^372^ In 2022, Li et al. developed Nvwa, a deep-learning-based strategy, which predicts gene expression and identifies conserved regulatory programs underlying cell types at the cross-species single-cell level.^373^ In the future, we also hope to find similar cross-species conservative regulatory elements in the aging process through development of machine learning algorithms.

The immune system has a remarkable ability to remember and respond to different stimuli and experiences, leading to heterogeneity in immunosenescence among individuals. This heterogeneity can result from differences in the type, dose, intensity, and temporal sequence of antigenic stimuli to which each person is exposed. To address this issue, Franceschi et al. proposed the concept of “immunobiography,” which considers the unique history of antigenic exposure that shapes an individual’s immune system over time.^374^ While immunobiography provides a comprehensive framework for understanding immune aging, it does not take into account other factors that can influence the accumulation of inflammation and the aging process, such as genetics and social factors. Therefore, a more holistic approach that considers multiple factors may be necessary to fully understand the complexities of immunosenescence and inflammaging.

Preventing and alleviating the diseases of aging and improving quality of life are the ultimate goals of aging research. Current anti-aging strategies include eliminating senescent cells, stem cell therapy, and organ transplantation, whose essence is anti-inflammatory. However, what came first, the aging (chicken) or the inflammation (egg)? The exact causality between inflammation and aging deserves further studies for efficient intervention of aging-associated diseases and enhancing well-being.
